# The development of the ScoPeO-Kids: A new measure of quality of life for children living in contexts of vulnerability

**DOI:** 10.1371/journal.pgph.0007247

**Published:** 2026-09-09

**Authors:** Johanne Higgins, Lise Poissant, Stephanie Legoff, Lise Archambaud, Aude Brus

**Affiliations:** 1 School of Rehabilitation, Université de Montréal, Montréal, Quebec, Canada; 2 Centre de recherche interdisciplinaire en réadaptation du Montréal métropolitain (CRIR), Institut Universitaire sur la réadaptation en déficience physique de Montréal, Montreal, Quebec, Canada; 3 Fédération Handicap International/ Humanity & Inclusion (HI), Lyon, France; ICDDR: International Centre for Diarrhoeal Disease Research Bangladesh, BANGLADESH

## Abstract

Access to valid and reliable quality of life measures is of the utmost importance when evaluating how effective interventions targeting vulnerable children are.. The aim of this project was to develop a measure to evaluate the outcomes of interventions that aim to create enabling environments for the development of young children and adolescents living in situations of vulnerability. A standard multi-phase approach integrating qualitative and quantitative methods was used. The identification of key domains, the generation of a pool of items and their response scale was done through a literature review, workshops and focus groups conducted with experts in the field and children living in situations of vulnerability and their parents. The instrument was administered to 150 children from each to pilot test the pool of items. Finally, Rasch analysis was performed on this item pool to verify psychometric properties. A 20-item version of a measure of QoL was created. This new measure will be further tested among another group of children living in communities receiving similar services to verify validity (known-groups) and reliability (test-retest).

## Introduction

Measuring Quality of life (QoL) has been acknowledged as a valid and important concept to capture the impact of a disease or particular condition on individuals as well as to determine the efficacy of interventions [1]. Improving QoL is particularly relevant and often a primary outcome for organisations that deliver interventions that aim to improve living conditions and ensure that the basic needs of vulnerable populations are met and respected. However, monitoring and assessing QoL as a performance indicator for projects and services deployed in various environments across various populations remains a challenge, especially for children. Access to QoL measures that are valid and reliable is just as crucial to assess the effectiveness of these interventions [2]. But identifying the ‘right’ measure is a complex task.

For some, QoL should integrate the subjective perception of an individual across all life domains, including physical and emotional health and his/her social context [1,3]. Others will express QoL as representing the gap between one’s perception and feelings towards his/her functioning and his/her perceived ideal functioning [4]. The World Health Organization (WHO) [5] refers to QoL as ‘an individual’s perception of their position in life in the context of the culture and value system in which they live and in relation to their goals, expectations, standards, and concerns’. The WHO integrates six dimensions into the construct of QoL: physical health, psychological health, independence level, social relations, environment, and personal beliefs.

The Australian QoL center defines another set of dimensions; health, material well-being, productivity, intimacy, security, role in the community and emotional well-being. In children, some will argue that QoL must also integrate the interaction between the child and his/her environment [6]. Others [4] state that emotions, social interactions, school, medication or treatments, cognition, activities, leisure/play, independence level, pain, perspective of the future, body image are concepts related to the QoL of children.

In this study, vulnerability is defined as a situation of significant exposure to threats or hazards that may limit the satisfaction of basic needs and access to fundamental rights, depending on individuals’ capacity to anticipate, cope with, or recover from these threats. Vulnerable children therefore include those living in poverty, social exclusion, conflict, displacement or disaster, and who may also present with a disability. QoL in vulnerable children often highlights the domains of poverty and poor education [7], anxiety levels [8] social and environmental factors. Mothers’ emotional well-being and the quality of children’s peer relationships were also strong predictors of the QoL of children living in refugee camps [9]. Low levels of psychological social support and poor resilience capacity among North Korean adolescent refugees were associated with higher risks of depression [10] which, in turn, could influence QoL.

Therefore, when developing a QoL measure for vulnerable children, there are several challenges to address. The first one being that most QoL measures for children are developed in industrialized or high-income countries. This raises great concerns because of the importance of cultural factors involved in the concept of QoL. If used in a different socio-cultural context from where they were initially developed, results may not be valid, as different cultures may not share the same definition of QoL [11]. Furthermore, several existing QoL measures for children are health-related, thereby imposing a QoL perspective that may differ from the one required to assess the QoL of vulnerable children [2]. Children’s physical and mental growth, as well as their emotional well-being, are deeply interconnected and influenced by a range of social, environmental, and psychological factors that should be assessed holistically in any QoL measure [12]. The conceptual frameworks shaping ScoPeO-Kids help identify and categorize domains and do not impose a strict structure. Domains concerning physical health, autonomy, and emotional well-being are informed by frameworks that emphasize functioning, capabilities and subjective well-being. Furthermore, Maslow’s hierarchy of needs is used to prioritize basic needs and safety as fundamental components of QoL in vulnerable groups.

Because QoL is multidimensional and specific to each context, the development of ScoPeO-Kids was based on a structured process aimed to reduce subjectivity in the selection of domains and items where the opinion of stakeholders—children, parents, and field professionals—were synthesized in a complementary way so as to achieve a balance among conceptual relevance, as well as ecological validity. Items were selected based on their consistency across groups, cultural relevance, and direct connection to children’s lived experiences. In contrast, ideas commonly found in QoL measures but not as relevant in humanitarian settings were not included. This approach ensured that the items chosen were based on shared priorities and not random or purely theoretical ones.The objective of this study was to develop a new measure of QoL for children aged between 5 and 18 years living in deprived social and economic environments such as refugee camps, slums or communities with a high prevalence of poverty. In the context of this study, vulnerable children are defined as those living in poverty, exclusion, conflict or disaster and that may present with a disability. It also includes internally displaced children, known as one of the most vulnerable people in the world, and a population reporting poor QoL [13].

This study was conducted in collaboration with Humanity and Inclusion (HI) an ‘independent and impartial non-governmental organization (NGO) working in situations of poverty and exclusion, conflict and disaster’ (https://www.hi.org/en/hi-network), as part of a project that aimed to help communities raise socially and emotionally healthy children in displacement settings, by creating safe opportunities for children to learn and develop their potential, whilst also having fun. The need for a tool capable of measuring the outcomes of such interventions on the QoL of children regardless of their gender, age or disability status was essential. The quality of life (QoL) for individuals in vulnerable populations can be significantly impacted by factors such as poverty, disability, and environmental conditions, making it essential to consider these contextual elements when designing and evaluating QoL measures [14].

## Materials and methods

### Ethics approval and consent to participate

Ethical approval to conduct this study was obtained from the Comité d’éthique de la recherche en santé of Université de Montréal (CERES-16–159-P). Signed consent was obtained from all participants of the focus groups and the survey. Signed consent from parents or a legal guardian/tutor was obtained for all children aged less than 14 years. All children aged 14 and over signed the consent form. All data collected during the developmental phases were anonymous, transmitted anonymously to the research team and kept on a secure server. Parents and children for all phases of the study were recruited between 20/03/2017 and 20/12/2018. Information on authorship and local and ethical engagement is provided in S1 Text.

A standard multi-phase approach integrating qualitative and quantitative methods was used [15,16]. The qualitative and quantitative phases that led to the development of the questionnaire are reported in this paper.

### Defining the purpose of the instrument and construct to be measured

Improving the QoL of NGO’s beneficiaries is a performance indicator often used to assess the outcomes of proposed intervention projects on the terrain. This led Humanity and Inclusion to develop the Score of Perceived Outcomes (ScoPeO), a multidimensional and dynamic QoL measure where adults express their perceptions/feelings in seven dimensions: physical and mental health, social and personal relationships, subjective well-being, basic needs, material well-being, perceived security and social participation and family [16]. The deployment of new projects targeting children in the sectors of education, rehabilitation or child health highlighted the need for a child-adolescent-specific version of the ScoPeO, the ScoPeO-kids to measure QoL of all children, regardless of their age or disability status.

No *a priori* definition of QoL was utilized. QoL, in this context, was explored in terms of needs, goals, expectations and concerns from psychological, social and physical perspectives, with each of these domains expressed in terms of functioning, capability or subjective well-being [5,17]. Regardless of the authors or literary source, QoL integrates health or health-related concepts such as pain and physical health. However, QoL is more than just health [18]. In an attempt to better understand the QoL of individuals with severe incapacity, Round and colleagues (2014) identified three particular concepts that have framed the development of HRQoL/QoL instruments, that is, functioning, capabilities and subjective well-being. Instruments, under the functioning framework, assess QoL based on how individuals perform certain activities. Under the capability framework, QoL is defined in terms of what people can do and be in their lives. Finally, instruments based on the subjective well-being approach place emphasis on a person’s own evaluation of whether life is good or not, brings happiness or sadness, etc. The models proposed by Round and colleagues [17] and Fagerlind schematisation [18] illustrate that being healthy and functioning normally contribute to both concepts of health and quality of life, while domains such as one’s living conditions, social network and attitude towards life contribute more to a person’s quality of life. Both were used as frameworks to support the reflexion behind the development of the ScoPeO-kids. They were completed by Maslow’s goals or needs perspective [19]. Drawing on Maslow’s hierarchy, the measure was developed on the premise that QoL among vulnerable children is closely linked to the satisfaction of basic needs. Basic needs such as food, shelter and security were also put forward by field experts from HI and the literature as major concerns to be addressed when working with children living in refugee camps and slums [20], directly informing their inclusion. Social and emotional well-being domains were also considered to reflect higher-level needs, conceptualized as dependent on, and interacting with, the fulfilment of basic needs.

#### Identification of key domains and generation of a pool of items.

The initial item generation combined inductive and deductive approaches. The deductive approach was based on a review of the literature on existing age-specific versions of QoL measures and to identify key factors to consider for the development of the new measure. The following key words: child*, pediatric, infant, adolescent, teenager, quality of life, well-being, measure, test, evaluation, assessment, rating, surveys and questionnaires were used to search the following databases: PubMed, Google scholar and Medline. A manual search was also conducted on the Can-Child web site (https://www.canchild.ca/fr). Sub-scales or sections’ labels on existing QoL measures were identified as domains contributing to the QoL concept.

The inductive approach was based on a qualitative phase involving children, parents and experts. First, a workshop with experts (HI personnel) was conducted to confirm domains and response scales that were identified in the literature review and to identify any missing domains. Secondly, focus groups were held with children and parents living in social and economic environments such as refugee camps, slums or communities with a high prevalence of poverty to explore the concept of QoL from a cultural and context-specific perspective. More precisely, participants were recruited from distinct settings: urban slums and more rural settings as well as refugee camps. Slums typically consisted of densely packed, improvised housing constructed from temporary materials [21]. Refugee camps were semi-permanent settlements hosting displaced persons fleeing conflict and persecution. The camps were characterized by restricted freedom of movement and reliance on humanitarian aid [22].

#### Workshop With HI.

A total of 18 HI personnel from Thailand, Bangladesh and Pakistan participated in a two-day workshop. Field experts were invited to review domains and response scales associated to QoL from existing literature, to generate new domains and to express, in their own words, how the psychological, physical, social, environmental and basic needs domains could be operationalized to capture the QoL of children living in refugee camps or slums.

#### Children focus groups.

A purposive sampling technique was used whereby HI site workers identified children within three age groups (5–7 years, 8–12 years and 13–18 years) who had the cognitive capacity and willingness to participate in discussions and were able to communicate verbally. Age groups were established based on recommendations from HI field experts and based on existing age-specific QoL measures for children. Camp-based HI personnel approached children and parents through their usual channels of communications. HI personnel were asked to recruit children who were representative of the camp’s population, with a mix of children with and without disabilities. Children and parents were identified and recruited through HI’s local field teams and partner organisations operating in the selected refugee camps, slums and host communities. Potential participants were approached through existing community structures, such as schools, community centers and program activities. Field staff were instructed to recruit children who were broadly representative of the local population served, including children with and without disabilities, and across age groups, and to ensure the inclusion of children with diverse functional profiles to reflect the heterogeneity of the population served by HI. Children with disabilities were eligible to participate provided they had the cognitive capacity and willingness to engage in group discussions and could communicate verbally. No exclusion criteria were applied based on the type or severity of disability.

In Thailand, focus groups were conducted in a closed setting in Burmese refugee camps in Mae Hong Son and Tak provinces, whereas in Bangladesh, they were conducted in closed and open environments, in a slum and a rural community outside Dhaka. Twelve focus groups attended by a total of 87 children were conducted in Thailand and Bangladesh. Four focus groups were conducted, comprising 33 parents (14 in Thailand and 19 in Bangladesh). In Thailand, all participating parents, except one, were women. In Bangladesh, 33% of participants were men. Characteristics of participants are presented in Table 1.

**Table 1:** Characteristics of Participants of the Focus Groups.

	Thailand			Bangladesh		
Participants	N	Mean Age (yrs)	Sex (F/M)	N	Mean Age (yrs)	Sex (F/M)
*Children*						
FG1: 5-7 y.o	6	6.2	4/2	6	6.8	5/1
FG2: 5-7 y.o	7	6.7	3/4	11	6.4	7/4
FG3: 8-12 y.o	5	10.2	3/2	10	8.5	8/2
FG4: 8-12 y.o	6	8.8	3/3	8	10.2	5/3
FG5: 13-18 y.o	7	14.7	4/3	8	13.4	7/1
FG6: 13-18 y.o	6	16.3	6/0	8	16.1	5/3
*Parents*						
FG1	7	46.8	6/1	9	38.9	6/3
FG2	7	36.0	7/0	10	41.8	6/4

FG; focus group, yrs; years; y.o; years old, F;female, M; male.

On average, focus groups (FG) were held in the morning and lasted between 60 and 90 minutes, with a short break and time for play. Parents were not allowed to be in the room with their children in order to favour openness from children. Interview grids with open-ended questions were developed for each age-specific FG. For example, participants were asked to discuss their day-to-day activities, their social relations (school, family, etc.), their psychological well-being (what made them proud, what made them sad, etc.). To facilitate discussion among participants, younger children were given a sheet with a variety of emoticons and were invited to select the emoticon that best expressed how they felt and were then invited to discuss their feelings. Photos (e.g., child crying, child with family, etc.) were also used as prompts for younger children (5–7 y.o) or if perceived to be useful to children of other age groups.

#### Parents focus groups.

Focus groups were conducted with parents of children living in refugee camps in Thailand and in a slum and a rural community in Bangladesh. Parents were recruited by HI personnel. Participants had to be able to express themselves verbally. Special attention was given to the selection of parents of children from different age groups. An interview guide specific to parents was developed and discussions focused on understanding factors affecting the QoL of their children.

All focus groups were held in the native language of participants. A translator was present to ensure adequate communication between participants and the animator. (i.e., questions were asked in English, translated to the participants and their responses were, in turn, translated back to the research team in the most timely manner as possible).

All focus group discussions were audio-recorded and the translator’s communications were transcribed verbatim. In light of the structure and goals of the focus groups (emergence of themes/domains), a thematic analysis approach was used, guided by the frameworks previously discussed and the FG interview guides (S2 Text). Two coders independently read and coded all verbatim. Emerging codes were assembled into themes that were then reviewed, discussed and refined by the two coders [23]. QDA-Miner software was used for the analyses.

### Selecting the items and the rating scales, identifying biases and assessing validity and reliability according to the Rasch model

A stratified sampling technique was used to recruit 150 children from each of the three age groups to pilot test the pool of items. Under the supervision of the research team, recruitment was performed by HI camp-based collaborators who invited all potential children in relevant communities (i.e., schools, play environments, community centres, etc.) using available and accessible means of communications (meetings, posters, etc.). Demographic information was collected by trained interviewers as part of the data collection process on paper. The questionnaire, originally developed in English, was translated into each of the appropriate languages (Karen and Burmese for Thailand; Bangla for Bangladesh and Urdu for Pakistan) for each of the countries and administered to children by HI-camp-based collaborators. Parents were asked to provide demographic information for children in the younger group of children. The questionnaire was initially developed in English and subsequently translated into the relevant local languages (Karen and Burmese in Thailand; Bangla in Bangladesh; Urdu in Pakistan). Translation was coordinated and supervised by HI’s local field teams in each country, who have extensive experience working with children in humanitarian and low‑resource settings. While a formal forward–backward translation procedure was not systematically documented, particular attention was paid to cultural and contextual appropriateness of item wording. To support cross‑language equivalence, cognitive debriefing interviews were conducted with children in each country to assess item comprehension, relevance and interpretation. Findings from the cognitive debriefing indicated that children across languages interpreted the items as intended, suggesting that the translations did not introduce a systematic interpretation bias.

Children with disabilities were included in the survey phase following the same criteria as other children. The recruitment process aimed to reflect the diversity of children receiving services, including those with different functional limitations. To help with participation, interviewer-led interviews and visual aids were used as needed.

### Initial psychometric analyses

Rasch analyses were performed with RUMM2030 software [24] using the Rasch partial credit model. These analyses were conducted to obtain a preliminary evaluation of the newly developed measure, focusing on item performance and evidence of unidimensionality.

Overall model fit and individual item fit statistics were evaluated using global fit statistics and individual item fit using fit residuals and chi-square statistics (S1 Table). The functioning of response categories was assessed by examining the ordering of response categories for each item. Unidimensionality was examined using principal component analysis (PCA) of the item residuals and was further tested using paired *t-*tests comparing person estimates derived from item subsets loading positively and negatively on the first residual component, with ≤5% significant *t*-tests taken to support unidimensionality. Monotonicity was assessed by examining the ordering of the response categories for each item and local item dependence (LID) was evaluated by inspecting residual correlations between pairs of items. Residual correlations exceeding the recommended threshold (>0.30) were considered indicative of response dependency.

Also, as there were concerns that children of different ages may not answer questions in the same manner as older children and that translation or culture may influence children’s response, an analysis of differential item functioning (item bias) allowed to investigate lack of invariance for age groups and country of origin. Reliability was assessed using the Person Separation Index (PSI), which reflects the ability of the measure to discriminate between individuals with different levels of Quality of Life. The PSI is analogous to Cronbach’s alpha and is interpreted similarly. Finally, targeting of the scale was examined by comparing the distribution of person locations with item threshold locations along the latent trait continuum.

#### Deciding on a final set of items using a cognitive approach.

Following the Rasch analyses and to support the final selection of items, a cognitive debriefing exercise was conducted by one HI personnel in each country. HI personnel from each country were asked to recruit 8–9 children capable of communicating verbally, representing the different age groups and gender. In total, 9 children from Pakistan (5–13 y.o; 7F:2M), 8 children from Bangladesh (7–14 y.o; 4F:4M) and 9 children from Thailand (6–17 y.o; 7F:2M) participated in the cognitive debriefing exercise. Cognitive debriefing interviews is a common process used in the design of a questionnaire, as it informs clarity, relevance and comprehension of items by the target population. In total, 25 children aged between 5 and 17 participated in the cognitive debriefing exercise. The process used was systematic across participants, i.e., following the gathering of some socio-demographic information, children completed the questionnaire through an interview with a trained interviewer and were then asked, for each item of the questionnaire, to answer the following questions: do you understand this item? Can you explain what it means? Are response options relevant to the item? Responses were then aggregated per country to identify any country-specific differences.

## Results

### Identification of key domains

Key themes emerging from the literature and the analyses of QoL tools for children included social/family relations, school, functioning, physical activity, health, daily activities, self-esteem/ emotional well-being. The safety of the environment was mentioned in a small number of instruments. At the end of the workshop with HI experts, several concepts organised into four domains contributing to the QoL of vulnerable children (see Table 2) were identified. Those became potential items that were then pooled under each domain and discussed so that a consensus across groups could be reached on the items to be retained.

**Table 2:** Operationalization of themes identified by HI experts.

Physical	Social	Basic needs	Environment
Health/ Feeling healthy	Freedom expressing opinion	Food	Good health services
Pain	Being listened to	Water	Access to health services
Skill development	Opportunities to learn	Shelter	Affordable health services
Physical mobility	Family	Sanitation	Cultural sensitivity
Autonomy	Presence of parents or siblings	Warm/heat	Sensitized parents and society
Self-care	Good parenting	Rest	Child labor
Safety	Gender equality		Gender equality
Support (Physical care)	Empowerment		Good parenting
Play	Absence of work		Formal/informal/inclusive education
Time to relax	Time to relax and play		Availability of schools
	Sports and leisure		Didactic material
	Social relationships		Safety and protection
	Interpersonal interactions		Child abuse
	Friends and peers		Individual needs
	Support playing with other children		Vocational training for persons with disabilities
	Autonomy		

To obtain the first version (Version 1) of the questions, which comprised 43 statements that were scored on a 5-point Likert scale from strongly disagree to strongly agree, information from focus groups was analyzed. Several themes were raised during the focus groups. Table 3 highlights those shared by parents and children, regardless of age and country. Children strongly expressed the importance of having friends to play with, talk or confide with. Restricted access to school and to friends by parents raised negative emotions such as anger or sadness among children. Being able to take one’s own decisions was particularly important for teenagers and acknowledged by parents as an important component of their child’s well-being, or QoL. For all children, regardless of age, feeling valued by parents was important as it was, for many, directly associated with some form of physical expression of love and appreciation. Except for the youngest group, confidence and optimism about the future appeared as factors influencing their well-being. Parents were, by far, more pessimistic and less confident in their child’s future QoL but all recognized it was important to encourage their child’s optimistic view of the future and confidence in their capacity to achieve their dreams. Pain and disability were expressed by all groups as negatively influencing one’s QoL, particularly through restricting school attendance, play with friends and autonomy. Young children experiencing being left alone at home for several hours or having to take their meals alone reported these situations as bringing sadness and anxiety. Finally, parents associated the safety of the environment as an important factor affecting the child’s capacity to participate in social activities and, hence, to favour his/her well-being. Finally, a majority of parents expressed how poverty affected their capacity to fulfil their child’s basic needs in terms of food. Being hungry was seldom reported by children as a factor affecting their well-being, although some expressed having difficulty staying attentive during morning classes.

**Table 3:** Themes Generated by Children and Parents.

Themes/Domains	Quotations
*Personal development*	
Knowledge development	“I like going to school, to read” (FGT1)‘I like school because I learn new things”(FGB2), ‘education is important” (FGP)
Learning new things/skills/creativity	“I like singing and dancing” (FGT3), “school is useful because it helps me develop new skills”, “important for children to shine” (FGP)
*Social well-being/belonging to a group*	
Being listened to	“I like to talk about my day at school to my mom”(FGB3), “my friends are important because we can share experiences with each other”(FGT3)
Having friends/Belonging to a group	“I like spending time with friends”(FGT1), “Doing sports with friends makes me happy”(FGB3), “Friends bring happiness to children” (FGP)“”
*Self-esteem*	
Being able to take own decisions	“I want to study but my parents want me to work, that makes me feel angry”,(FGB3) “Even if I don’t like something I cannot say no, I have to do it”(FGT2)
Feeling useful/valued	“I like when I help my mother with housework”*(FGT1) “when my mom is sick I do the cooking, it brings her some relief” (FGB2) “I help my mom when my father is away, I know she’s happy about it because she smiles at me”,(FGT2) “It is important to encourage our children when they have good results’ (FGP)
Having confidence in self	“I try the best until I reach my goal”,(FGT3) “I don’t have enough skills to be satisfied” (FGT3)
*Physical health*	
Being healthy/pain free	“I am sad when I am sick because I cannot play with my friends, I cannot go to school”(FGT2) “Disabled children should have the same opportunities as non-disabled children” (FGP)
*Emotional well-being*	
Being hopeful	“I want to do something good in my life” (FGT3), “ I don’t know what will happen, the situation is very unsure” (FGPT), “Children have their own ambitions, it is important to help them”(FGPB)
Being happy/sad	“playing with friends makes me happy” (FGT2) “playing makes children happy”(FGPT)
Living in a safe/trustful home	“my parents scold me if I don’t do the chores”(FGB1), “sometimes my father scolds me if I play and I should not be playing”(FGT
Feeling loved	“My parents smile at me”(FGT1) “ my parents discuss with me about my day at school”,
Taking meals with family	“taking meals with my family makes me happy”(FGT1), “I like when we all go to our village house “ (FGB1)
Feeling lonely	“I feel sad when I am alone at home” (FGB1), “my mom works outside and I don’t see her often, that makes me sad”(FGB2)
*Environment*	
Feeling safe	“My parents don’t let me play outside”(FGT3) “if the playground is too far, my parents will not let me go” (FGB3) “We are trying to make things better and safer for the girls” (FGPB)

FG: Focus group; B: Bangladesh; T: Thailand; 1: 5–7 y.o; 2: 8–12 y.o; 3:13–17 y.o; P:Parents

### Generation of a pool of items and response options

The research team and HI experts discussed and compared the findings from the workshop and the focus groups to obtain a revised set of domains and themes after comparing findings. This led to 19 themes being chosen and grouped into three domains (Table 4). For each theme, existing QoL measures were reviewed to identify potential statements/items. Version 1 of the questionnaire was then developed.

**Table 4:** Combined set of domains and themes from HI experts, children and parents.

Physical domain	Psychological domain	Social domain
Autonomy in self-care Play Feeling healthy Pain Being hungry.	Being listened to Being appreciated by others Feeling useful Having confidence Making own decisions Knowledge development/ learning new skills Feeling equal to others Feeling happy Feeling sad Having balance between play/leisure and work	Belonging to a group Having social support Living in a loving environment Taking meals with family.

Version 1 comprised 43 statements that were scored on a 5-point Likert scale from strongly disagree to strongly agree – a higher score representing a higher level of quality of life. It was decided to deliberately have more than one statement per theme to test its relevance and to provide capacity to select the item that would best perform during the quantitative phase. Smiley faces were added to the wording scale to facilitate utmost of the response options for children in the younger age group and those who were unable to read as confirmed by HI personnel. The use of smiley faces was based on recommendations/preferences expressed during the workshop with HI personnel and their prevalence in existing QoL measures for young children. All items were phrased so that the crying face would be associated with the strongly disagree option and the smiley face with the strongly agree option.

### Selecting the items and the rating scales, identifying biases and assessing reliability according to the Rasch model

Overall and across Thailand, Bangladesh and Pakistan, 574 children consented to participate. All 574 questionnaires were completed and used for the analyses. Table 5. presents demographic and clinical characteristics of study participants.

**Table 5:** Demographic Characteristics of Survey Participants (n = 574).

Participant characteristic	N (%)
**Age**, mean (SD)	10,7 (3.7)
5-7	195 (34)
8-12	177 (31)
13-18	201 (35)
**Gender**,	
male	302 (53)
**Country**	
Thailand	207 (36)
Bangladesh	176 (31)
Pakistan	191 (33)
**Status** *	
Refugee	315 (55)
IDP	3 (0.5)
Host Communities	252 (44)
Other/missing	4 (1.1)
**Living with** *	
Parents	551(96)
Grand parents	11(2)
Other	11(2)
**Receiving aid services** *	
No	317(55)
Yes	247(43)
**Dwelling** *	
Village	254 (44)
Refugee camp	310 (54)
Other/missing	10 (2)
**Main activity**	
Preschool/kindergarten	45 (8)
School	440(77)
Work	14(2)
Neither	68(12)
Missing	7(1)

*Percentages may not add up to 100 due to rounding.

IDP: Internally Displaced Person.

### Overall and individual item fit to the Rasch model

Rasch analysis was used to examine if the items functioned as expected to measure QoL. A good fit to the model indicates that items are ordered consistently along the QoL trait and that children with higher QoL are more likely to answer positively to higher response categories. The first run of the Rasch analyses with all 43 items and 574 children demonstrated misfit to the Rasch model producing a significant item-trait interaction (χ2: 1182.96; probability: p < 0.05). This result suggests that some items do not contribute to measuring QoL across children uniformly.

A total of 16 items did not fit the model. Eleven items had fit residuals outside the ± 2.5 range and 13 items had chi-square or fit residuals p < 0.001 (bonferoni adjusted), indicating a difference between observed responses and the expectations of the Rasch model. Fit residuals reflect the extent to which each item conforms to the model; large positive or negative values indicate that an item may be measuring something different from QoL or that they function inconsistently across children.One person had an extreme score and was excluded from the analyses, resulting in a sample of 573 children. PCA of the item residuals indicated that the first residual component had an eigenvalue of 3.2 and accounted for approximately 8% of the residual variance, followed by a second residual component with an eigenvalue of 2.5 explaining 6% of the variance. In Rasch analysis, unidimensionality implies that all items measure a single latent construct; residual components with eigenvalues greater than 1 may indicate the presence of additional dimensions beyond the primary QoL trait.

Although a dominant Rasch dimension was observed, accounting for less than 10% of the variance, the presence of residual components with eigenvalues greater than 1 suggested potential multidimensionality. Further assessment of unidimensionality using paired *t*-tests following PCA of residuals (extreme persons excluded; *N* = 566) indicated that only 17.84% of item-set comparisons met the ≤ 5% criterion, suggesting multidimensionality. Together, these findings suggest that the initial set of items captured more than one underlying dimension, warranting further investigation.

### Response categories

Response category were examined to determine whether children used the response options in an ordered way. Disordered thresholds indicate that children have difficulty distinguishing between adjacent response categories, suggesting that the response scale may be too complex.

**Fig 1:**
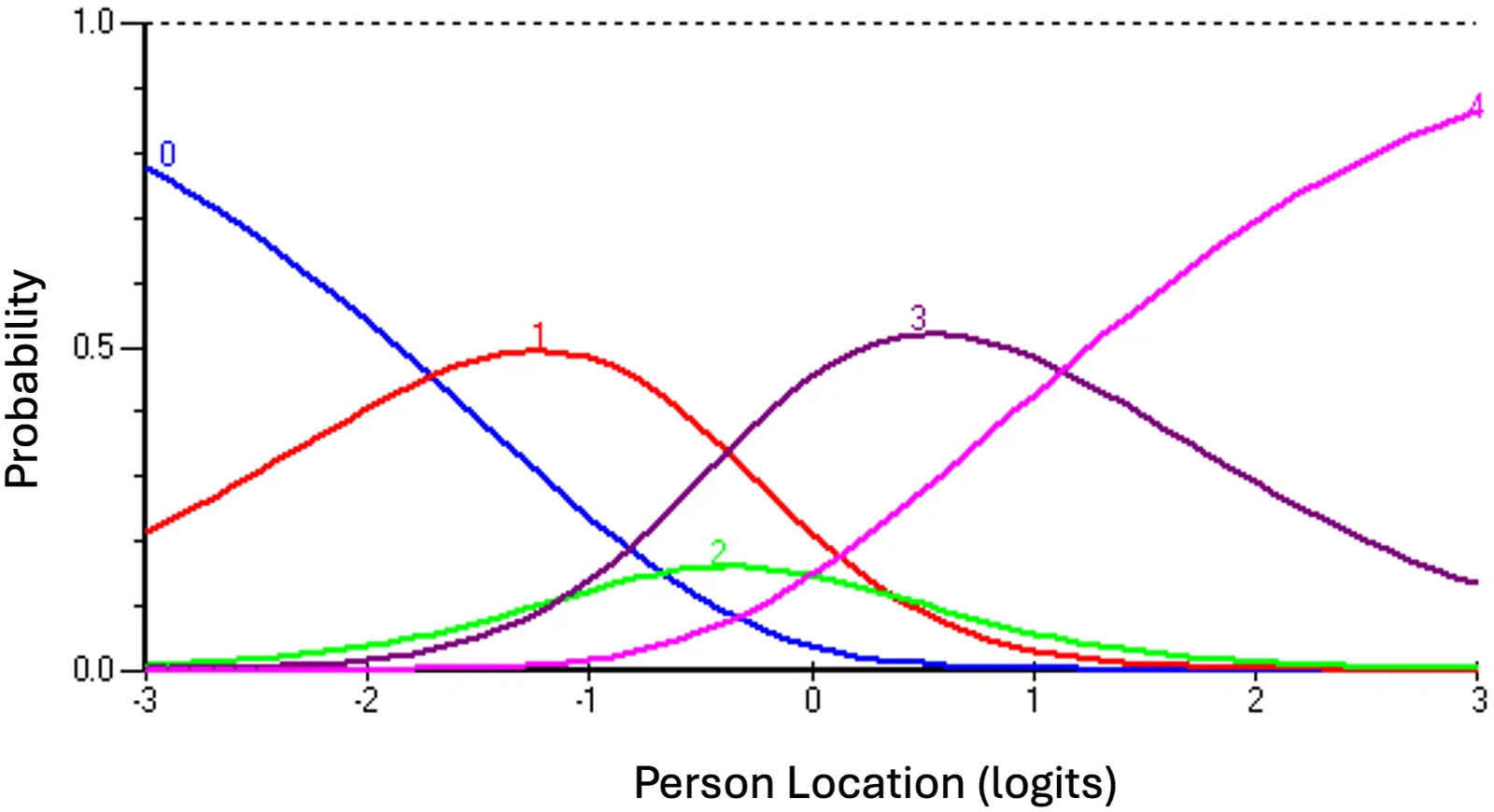
Example of Category probability curves showing disordered thresholds (Item #11: I can eat, dress and wash by myself).

Examination of category probability curves revealed that all 43 items displayed disordered thresholds**,** indicating that the original five-category response scale was not functioning as intended (see Fig 1). Response category 2 was consistently underused across items. After collapsing categories to form a four-point response scale, thresholds became ordered for all items, supporting monotonicity of the revised response format.

### Differential item functioning (DIF)

Differential item functioning (DIF) analysis was conducted to assess whether items were interpreted or answered differently by subgroups of children with the same level of QoL. Evidence of DIF suggests potential item bias related to factors such as language, culture or context rather than true differences in QoL.

Rasch analyses indicated that two items *I feel good about doing chores at home* and *I have someone who listens to me when I need to talk* behaved or were answered differently depending on the country where the survey was administered, indicating that the questions were ‘biased’ potentially because of a translation or a cultural issue. To verify, the questions were re-translated into the language of each individual country and back-translated into English. Changes to the wording of the questions were made when warranted. Results of the Rasch analyses indicated no biases with regards to age for any of the items present.

### Local item dependence

Local item dependence occurs when responses to one item are directly influenced by responses to another item, rather than by the underlying latent trait alone. High residual correlations between items may indicate redundancy or overlap in item content. Residual correlation analysis identified four item pairs with correlations exceeding 0.30 (range: 0.306 to 0.520), suggesting the presence of local item dependence.

### Reliability

Scale reliability, assessed using the Person Separation Index (PSI)**,** was 0.92, indicating excellent reliability for group-level comparisons and demonstrates that the ScoPeO-Kids can effectively discriminate between children with different levels of quality of life.

### Targeting

Targeting refers to the degree of alignment between the difficulty of the items and the quality of life levels of the respondents. Good targeting indicates that the items adequately cover the range of QoL experienced by the persons in the sample. Targeting analysis showed that the mean person location was 0.95 logits, which is higher than the mean item location (0 logits), indicating that children, on average, demonstrated a higher quality of life than that targeted by the items. The person–item threshold map confirmed the presence of a slight ceiling effect, as some respondents were located beyond the most ‘difficult’ item. No floor effect was observed, as no respondents were located below the ‘easiest’ item.

Difficult items are ones for which a higher level of Quality of Life is required to endorse higher response categories.

### Transformation of the questionnaire

Following the initial Rasch modeling of the 43-item Scopeo-Kids, further investigation was undertaken to determine if the measure could be modified to improve the fit to the Rasch model. First, the response options were modified. Option 2 was collapsed with option 1, leading to a four-point scale. None of the items displayed disordered thresholds after this transformation.

However, a few items still did not fit the model. These were removed iteratively based on misfit statistics but also on the importance of the specific item within the questionnaire (i.e., the presence of similar items, the significance of the question, etc.) as well as the cognitive debriefing that followed. The unidimensionality of the final 20-item model was supported by the principal component analysis of the residuals. Indeed, the first component explained less than 10% of the variance, confirming that all the information in the data is explained by the latent measure [23].

### Results from the cognitive debriefing

A total of 13 items were revised by the research team to address potential clarity/comprehension issues. Items were also revised with the underlying aim of reducing the number of items so specific attention was given to items measuring similar concepts. A frequency scale was tested and was found to be well understood by the majority of children. The use of circles of different sizes as a visual support for the frequency scale was also appreciated and understood by all.

A final set of 20 items was kept. All items were revised so that the wording would fit a four-point frequency scale (never, not often/a little, often/a lot, always) and to ensure that domains were assessed by a minimum of two items. The final 20-item questionnaire (S3 Text) covers 5 domains; physical well-being (3 items), safety (2 items), autonomy and self-realization (5 items) and social well-being (5 items).

## Discussion

This paper describes the qualitative and quantitative phases leading to the development of the ScoPeO-kids, an evaluative measure of QoL for children living in contexts of vulnerability. The study illustrates the feasibility and added value of combining participatory qualitative methods with modern psychometric approaches when measuring complex constructs such as QoL in underserved populations. The close collaboration and implication of HI, the main knowledge user, is a major strength of this research project, enhancing the contextual relevance of the measure and increasing the likelihood of future uptake in practice. Best practices in measurement highlight the importance of identifying domains based on their perceived relevance by the target population and/or experts [25]. Our project incorporated both perspectives. HI expert opinions and their knowledge and understanding of their country, culture and population’s services needs facilitated guidance in the development of focus group discussion guides and in the interpretation of the qualitative data.

Our focus groups reached 87 children and 33 parents from different living conditions and countries. The large sample and the heterogeneity of individuals who were given the opportunity to express the significance of QoL for themselves or their children support the content validity of the proposed instrument and meet best practices in measurement [26–28]. The fact that we had parents of children with severe disabilities who participated in the parents’ focus groups and the presence of HI experts throughout the research process strongly supports the inclusiveness of the ScoPeO-kids and its capacity to capture QoL of all children regardless of the presence or not of a disability.

At first, the ScoPeO-kids comprised 19 themes organized under three large domains, physical, psychological and social. While several themes reached consensus among participants and were readily incorporated into this version of the instrument,some themes, for example, being hungry, having a balance between play/leisure and work, taking meals with family or living in a loving environment led to extensive discussions between members of the research team and selected HI experts. These discussions highlight the inherent challenges of measuring QoL across diverse cultural and socio-economic contexts. Some items (e.g., living in a loving environment) were felt to be culturally sensitive and to lead to potential response biases. Others (e.g., taking meals with family), were considered less important by HI experts but had been strongly expressed by children. In these situations, the relevance of an item (or theme) for children and /or their parents always took precedence over the opinion of HI experts and led to its inclusion in this version of the tool, reflecting the child-centered paradigm underpinning the development of the ScoPeO-kids. Conversely, items (ex. having balance between play/leisure and work) that were seen relevant by a minority of children and parents but that were strongly recommended by HI experts were also included in this version. An inclusive approach was preferred over a restrictive one, with the assumption that the quantitative phase would provide further data to support the selection of the final set of items. This strategy illustrates the role of psychometric testing as a decision-making tool to balance inclusivity with psychometric quality of the measure.

It is interesting to note that two domains (safety and autonomy/self-realization) appear to be specific to children living in contexts of vulnerability, such as refugee camps, slums or low socio-economic communities, supporting the need for the ScoPeO-kids tool as those were not seen among reviewed QoL measures. This finding supports the need for a specific measure of QoL and suggests that commonly used QoL frameworks may not fully capture the specificities of children living in contexts of vulnerability, as commonly used pediatric QoL instruments may assess safety and autonomy implicitly or indirectly. Maslow’s hierarchy showed how basic needs like having enough food and feeling safe affect how children living in vulnerable situations think about theirQoL. The ScoPeO-Kids domain structure combines established QoL frameworks with the real-life experiences of vulnerable children, strengthening its conceptual basis.Rasch analysis results informed subsequent refinements of the instrument, including modification of the response scale and removal of problematic items, thereby strengthening its measurement properties. Overall model misfit, multiple misfitting items, and evidence from residual analyses suggested that the assumption of unidimensionality was not fully met. Response category analyses indicated that the original five-category scale did not function as intended, with threshold disordering resolved by collapsing categories, supporting a four-point response format. The scale demonstrated excellent reliability, indicating that items were able to discriminate between different levels of QoL, although it should be interpreted with caution, as it may be inflated due to the multidimensionality. The targeting of the children is acceptable, although ‘harder’ questions, for which the children would need a higher QoL to answer positively would be helpful to eliminate the ceiling effect and improve targeting. None of the items displayed differential items functioning with regard to age and gender or country. Overall, these findings support continued item refinement and structural optimization to strengthen the measure’s psychometric properties and support future validation.

Based on the results of the Rasch analysis and cognitive debriefing, items considered as problematic were removed from version 1 of the questionnaire. This led to a 20-item version of the questionnaire covering 5 domains (physical well-being, emotional well-being, safety, autonomy/self-realization and social). Although physical well-being, emotional well-being and social domains are common domains of numerous generic or specific children’s measures of QoL or HRQoL [2,29,30], and known predictors of QoL among children [31] their conceptualization is expressed through items such as having enough food to eat or taking meals with family that are specific to children targeted by the tool. Our study also highlighted the need to integrate a safety domain to capture the potential impact of violence on the QoL of children [32], a distinctive aspect of the ScoPeO-kids over other generic measures. Anxiety and stress often reported as factors affecting QoL among refugees [8] are captured through different items within emotional well-being and the autonomy/self-realization domains. This study supports the importance of operationalizing QoL in light of the population’s specific needs or characteristics. As such, the ScoPeO-kids brings a unique and distinct contribution to the array of generic QoL measures for children. The targeting analyses indicated a slight ceiling effect, with some children presenting levels of QoL higher than those targeted by the most ‘difficult’ items. This suggests that, in subgroups of children with relatively higher baseline QoL, the instrument may have reduced sensitivity to detect small improvements over time. Given that the ScoPeO-Kids was designed for use in settings where the baseline QoL is often poor, the ceiling effect is not likely to significantly limit its ability to respond in intervention evaluations for children with low to moderate QoL.This study has limitations, the main one being the use of a translator throughout the focus group discussions with children and parents. It was impossible for the two researchers to control the quality of the translation of questions asked to participants and similarly to the responses provided by the participants. Also, the qualitative phase took place in only two countries, limiting the generalizability of our results and suggest further validation in a variety of different settings and countries would be advised.

Language was a strong barrier to timely interactions between the research team and participants. Several focus groups were conducted in an open environment with little or no control of distractors. This was particularly challenging for the younger groups (5–7 y.o.) but despite suboptimal environments, participants remained focussed and dedicated to the group discussions and, most importantly, free to express themselves. Another limitation relates to the response scale, which combines frequency- and intensity-related descriptors within single response options (e.g., “not often / a little,” “often / a lot”). Although this format was intended to reflect respondents’ overall perceived burden of an experience, it may introduce ambiguity in cases where an event occurs infrequently but with substantial impact, or frequently with minimal impact. While preliminary qualitative work suggested that respondents were generally able to provide a coherent global judgment, future research may require exploration of alternative response formats. Also, participant recruitment through partner organisations may have introduced selection bias, as children not engaged in community services or programs could have been less likely to be included.Finally, during the qualitative phase, participants had to be able to communicate verbally. Children and parents with severe hearing, attention or communication disabilities were excluded. Participants were thus not fully representative of the population receiving services from HI. Finally, translations of the questionnaire were supervised by experienced field teams and supported by cognitive debriefing. However, the absence of a formally documented forward–backward translation process represents a limitation; future studies could further strengthen cross‑cultural equivalence by incorporating standardized translation protocols.

In conclusion, this version will be further tested among another group of children living in communities receiving services of HI to verify validity (known-groups) and reliability (test-retest). The ScoPeO‑Kids is intended for group‑level program evaluation, with total and domain scores used to describe QoL profiles and monitor change over time, rather than for individual‑level interpretation, with higher scores representing better QoL. As the measure was developed using Rasch modeling, summed scores may be treated as interval‑level measures, allowing comparison of mean scores across time points or between groups. Domain scores may be useful for identifying which dimensions of QoL are most affected or responsive to change. Targeting analyses suggest the ScoPeO-Kids is most informative for populations with low to moderate baseline QoL, consistent with use in contexts of vulnerability.

## Supporting information

S1 Table The 43 original items and their Rasch measurement properties. (DOCX)

S1 Text Inclusivity in global research questionnaire. (DOCX)

S2 Text Focus groups Interview guide. (PDF)

S3 Text Scopeo-Kids questionnaire. (PDF)

## References

[1] Eiser C, Morse R. The measurement of quality of life in children: past and future perspectives. J Dev Behav Pediatr. 2001;22(4):248–56. doi: 10.1097/00004703-200108000-00007 11530898

[2] Clarke S-A, Eiser C. The measurement of health-related quality of life (QOL) in paediatric clinical trials: a systematic review. Health Qual Life Outcomes. 2004;2:66. doi: 10.1186/1477-7525-2-66 15555077 PMC534785

[3] De Civita M, Regier D, Alamgir AH, Anis AH, FitzGerald MJ, Marra CA. Evaluating health-related quality-of-life studies in paediatric populations. PharmacoEconomics. 2005;23(7):659–85. doi: 10.2165/00019053-200523070-00003 15987225

[4] Davis E, Waters E, Mackinnon A, Reddihough D, Graham HK, Mehmet-Radji O, et al. Paediatric quality of life instruments: a review of the impact of the conceptual framework on outcomes. Dev Med Child Neurol. 2006;48(4):311–8. doi: 10.1017/S0012162206000673 16542522

[5] World Health Organization. Measurement of Quality of Life in Children. London, UK. 1994.

[6] Lou C, Anthony EK, Stone S, Vu CM, Austin MJ. Assessing child and youth well-being: implications for child welfare practice. Evid Child Welf Pract. 2013;:91–133. 10.1300/J394v05n01_05 19064446

[7] Alduraidi H, Waters CM. Health-related quality of life of Palestinian refugees inside and outside camps in Jordan. Nurs Outlook. 2017;65(4):436–43. doi: 10.1016/j.outlook.2017.05.007 28622883

[8] Al-Smadi AM, Tawalbeh LI, Gammoh OS, Ashour AF, Alshraifeen A, Gougazeh YM. Anxiety, stress, and quality of life among Iraqi refugees in Jordan: a cross sectional survey. Nurs Health Sci. 2017;19(1):100–4. doi: 10.1111/nhs.12323 28058754

[9] Almqvist K, Broberg AG. Mental health and social adjustment in young refugee children 3 1/2 years after their arrival in Sweden. J Am Acad Child Adolesc Psychiatry. 1999;38(6):723–30. doi: 10.1097/00004583-199906000-00020 10361791

[10] Park S, Lee M, Jeon JY. Factors affecting depressive symptoms among North Korean adolescent refugees residing in South Korea. Int J Environ Res Public Health. 2017;14(8):912. doi: 10.3390/ijerph14080912 28805719 PMC5580615

[11] Wee HL, Lee WWR, Ravens-Sieberer U, Erhart M, Li SC. Validation of the English version of the KINDL generic children’s health-related quality of life instrument for an Asian population--results from a pilot test. Qual Life Res. 2005;14(4):1193–200. doi: 10.1007/s11136-004-2957-2 16041914

[12] Nazari J, Jafari K, Chegini M, Maleki A, MirShafiei P, Alimohammadi A, et al. Physical and mental growth and development in children with congenital hypothyroidism: a case-control study. Orphanet J Rare Dis. 2021;16(1):393. doi: 10.1186/s13023-021-02017-7 34556143 PMC8461833

[13] Getanda EM, Papadopoulos C, Evans H. The mental health, quality of life and life satisfaction of internally displaced persons living in Nakuru County, Kenya. BMC Public Health. 2015;15:755. doi: 10.1186/s12889-015-2085-7 26246147 PMC4527222

[14] Kazemi Karyani A, Rezaei S, Karami Matin B, Amini S. Poor health-related quality of life in Iran: decomposition analysis of affecting factors. IJHRH. 2019;12(1):28–37. doi: 10.1108/ijhrh-05-2018-0036

[15] Streiner DL, Norman GR, Cairney J. Health measurement scales: a practical guide to their development and use. Oxford University Press. 2024.

[16] Brus A, Duboz P, Lefebvre C, Legoff S. Mesurer la qualité de vie, la sécurité et la participation sociale et familiale des bénéficiaires des projets: L’outil ScoPeO. 2014.

[17] Round J, Sampson EL, Jones L. A framework for understanding quality of life in individuals without capacity. Qual Life Res. 2014;23(2):477–84. doi: 10.1007/s11136-013-0500-z 23975376 PMC3967061

[18] Fagerlind H, Ring L, Brülde B, Feltelius N, Lindblad AK. Patients’ understanding of the concepts of health and quality of life. Patient Educ Couns. 2010;78(1):104–10. doi: 10.1016/j.pec.2009.05.016 19560893

[19] Simons JA, Irwin DB, Drinnien BA. Maslow’s hierarchy of needs. 2009;9(2009):222.

[20] Lonn MR, Dantzler JZ. A practical approach to counseling refugees: applying Maslow’s hierarchy of needs. J Couns Pract. 2017;8(2).

[21] Intesar A, Parvez MS. Living with vulnerability: Triple burden through the eyes of urban slum women in Bangladesh. Social Sciences & Humanities Open. 2024;10:101014. doi: 10.1016/j.ssaho.2024.101014

[22] Hou T. Humanitarian aid practices on the Thai-Myanmar border after the coup: beyond depoliticization and inequality. Int J Humanitarian Action. 2024;9(1). doi: 10.1186/s41018-024-00159-4

[23] Miles MB, Huberman AM. Analyse des données qualitatives. De Boeck Supérieur. 2003.

[24] Andrich D, Sheridan BE, Luo G. RUMM2030. Perth: RUMM Lab Pty Ltd. 2010.

[25] Carpenter S. Ten steps in scale development and reporting: a guide for researchers. Communication Methods and Measures. 2017;12(1):25–44. doi: 10.1080/19312458.2017.1396583

[26] Stewart JL, Lynn MR, Mishel MH. Evaluating content validity for children’s self-report instruments using children as content experts. Nurs Res. 2005;54(6):414–8. doi: 10.1097/00006199-200511000-00008 16317363

[27] Lasch KE, Marquis P, Vigneux M, Abetz L, Arnould B, Bayliss M, et al. PRO development: rigorous qualitative research as the crucial foundation. Qual Life Res. 2010;19(8):1087–96. doi: 10.1007/s11136-010-9677-6 20512662 PMC2940042

[28] Detmar SB, Bruil J, Ravens-Sieberer U, Gosch A, Bisegger C, European KIDSCREEN group. The use of focus groups in the development of the KIDSCREEN HRQL questionnaire. Qual Life Res. 2006;15(8):1345–53. doi: 10.1007/s11136-006-0022-z 16826436

[29] Hullmann SE, Ryan JL, Ramsey RR, Chaney JM, Mullins LL. Measures of general pediatric quality of life. Arthritis Care Res. 2011;63(11S):S420-30. 10.1002/acr.20637 22588762

[30] Anthony EK, Vu CM, Austin MJ. TANF child-only cases: identifying the characteristics and needs of children living in low-income families. Journal of Children and Poverty. 2008;14(1):1–20. doi: 10.1080/10796120701871231

[31] Barthel D, Ravens-Sieberer U, Nolte S, Thyen U, Klein M, Walter O, et al. Predictors of health-related quality of life in chronically ill children and adolescents over time. J Psychosom Res. 2018;109:63–70. doi: 10.1016/j.jpsychores.2018.03.005 29580563

[32] Montgomery E. Long-term effects of organized violence on young Middle Eastern refugees’ mental health. Soc Sci Med. 2008;67(10):1596–603. doi: 10.1016/j.socscimed.2008.07.020 18755530

