# Supplementary material for: The development of the ScoPeO-Kids: A new measure of quality of life for children living in contexts of vulnerability
Source: PLOS Glob Public Health. 2026 Sep 9;6(9):e0007247. doi: 10.1371/journal.pgph.0007247 (PMC13557343; doi:10.1371/journal.pgph.0007247)
Supplement: Supplementary file 1 [file pgph.0007247.s003.pdf]

Appendix

Focus groups Interview guide - Children 5–7

Introduction

Hello everyone and thank you for being with us this morning! My name is Lise Poissant, and I am here today with my colleagues Johanne Higgins and Caline El Khoury. We come from Canada and we are here today to learn more about you. We want to find out what makes you happy or sad, how you spend your days and other things.

We will be together for about one hour (one hour and a half for older groups). We will take a break at some point but if you are tired or need to go to the toilet, just let us know and we will stop. We really want everyone to talk so don't be shy. There are no good or bad responses.

We would like you to raise your hand when you want to talk (like at school). This will greatly help us. We also need you to speak loud because we are recording you. If you can say your name before you talk, we'll appreciate.

- Can each of you say his/her name.
- Start with ice-breaker game: Cheryl

Emoticons and ice-breaker

Start by explaining emoticons.

Ice-breaker question: How are you feeling today? Are you in a good mood? Put your finger on the face that describes how you feel today.

ACTIVITIES

1) School

HOW DO YOU FEEL WHEN YOU ARE IN SCHOOL? Put your finger on the face….

- Do you like school? Is school fun? Why is it fun?
- What is it that you like at school? What don't you like?
- Do you find school easy? Why? Can you explain?

---

2) Play/leisure

- Do you have friends?

HOW DO YOU FEEL WHEN YOU ARE PLAYING WITH YOUR FRIENDS? Put your finger on the face….

- What kind of games do you play with your friends?
- Where do you play? Do you go there often? Are there places you would like to go and play but your parents don't let you?

WHY DO YOU THINK THIS CHILD IS PLAYING BY HIMSELF? Photo 1: enfant en train de jouer….

- Do you sometimes play by yourself like the little boy? When was the last time you played by yourself?

HOW DO YOU FEEL WHEN YOU ARE PLAYING ALONE? Put your finger on the face…. Can you explain (can you tell why you chose this face?)

---

3) Work/chores

- Do you work? Do you help in the house?
- Do you like it? How does it make you feel? Put your finger on the face…. Can you explain?

SOCIAL RELATIONS/FAMILY

HOW DO YOU FEEL WHEN YOU ARE WITH YOU FAMILY? Put your finger on the face….Can you explain?

WHAT DO YOU THINK THIS FAMILY IS GOING TO DO? (Photo de la famille)

- When was the last time you played with your parents? Mom and dad?
- When was the last time you had a meal with your parents? Mom and dad?

WHEN YOU THINK ABOUT YOUR MOM, HOW DO YOU FEEL?

WHEN YOU THINK ABOUT YOUR DAD, HOW DO YOU FEEL?

- Tell us about the last time your parents punished you?
- What happens when you disobey your parents?

HOW DOES THIS MAKE YOU FEEL?

BREAK

BREAK: Drawing of the face – and snacks

- Are you sometimes alone at home? HOW DOES THIS MAKE YOU FEEL?
- Are you sometimes scared? what do your parents do?

PHYSICAL HEALTH

- Are you able to run?

THINK OF DAYS WHEN YOU ARE SICK AND CANNOT RUN OR PLAY BALL WITH YOUR FRIENDS. HOW DOES THIS MAKE YOU FEEL?

DO YOU THINK THIS BOY CAN RUN? HOW DO YOU THINK HE FEELS? (Picture of boy in wheelchair)

PSYCHOLOGICAL HEALTH/WELL-BEING

WHY DO YOU THINK THIS CHILD IS SMILING? Picture of child smiling

WHY DO YOU THINK THIS CHILD IS SAD? Picture of child sad

DO YOU SOMETIMES FEEL ANGRY? CAN YOU TELL US THE LAST TIME YOU FELT ANGRY? Show angry emoticon

---

Focus groups Interview guide - <u>Children 8 and older (red questions are for 13 and older)</u>

Introduction

Hello everyone and thank you for being with us this morning! My name is Lise Poissant, and I am here today with my colleagues Johanne Higgins and Caline El Khoury. We come from Canada and we are here today to learn more about you. We want to find out what makes you happy or sad, how you spend your days and other things.

We will be together for about one hour (one hour and a half for older groups). We will take a break at some point but if you are tired or need to go to the toilet, just let us know and we will stop. We really want everyone to talk so don't be shy. There are no good or bad responses.

We would like you to raise your hand when you want to talk (like at school). This will greatly help us. We also need you to speak loud because we are recording you. If you can say your name before you talk, we'll appreciate.

- Can each of you say his/her name.

Ice-breaker question

If you were an animal, what animal would you be?

How are you feeling today? Are you in a good mood? What makes you in a good mood?

Activities

- Let's talk about what you do during the day. Can you tell us two things or activities that you really like doing?

School

- Do you like school? What is it that you like at school? What don't you like?
- Do you sometimes have trouble paying attention in school? Why?

- Do you find school easy? Can you explain? Do you find school useful? How is it useful?
- Are you satisfied with your results at school? Do you think your parents are proud of your schoolwork? Do you talk with them about school?

Play/leisure

- Do you like playing outside? Can you explain what you like and don't like?
- Think about the last time you played by yourself? Did you enjoy it? Why?
- Where do you play? Do you go there often? Are there places you would like to go and play but your parents don't let you?

Work

- Do you work? Do you help in the house? Do you like it? What makes you like it? or not like it?

If you could eliminate one thing from your daily activities, what would it be.

If you could learn any skills, what would it be?

Social relations

Family

- Let's talk about your family. Do you have brothers and sisters? What do you like doing with your family? Your parents? Your brothers and sisters? (examples, go to Temple together? Play together?) Tell us about the last time you had fun with your brothers and sisters? Tell us about the last time you played or had a meal with your parents? Was it fun?
- When you think about your mom, how do you feel? And about your dad?
- Do you quarrel at home with your parents or siblings? Can you tell us about the last you time you quarrelled? How does it make you feel?
- Do you talk about your day with your parents (about school or your friends)?
- Tell us about the last time your parents treated you unfairly or restricted you in what you wanted to do.
- When you are scared, what do your parents do? (think of a night when you were scared). Do you feel protected?

- Do you think your parents worry about you? Can you explain?

BREAK

BREAK: Drawing of the face – and snack

Friends

- Do you have friends? What do you usually do with your friends?
- Can you confide in your friends? Do you feel supported by your friends?
- Do you quarrel with your friends? Can you tell us about the last you time you quarrelled? How did you feel?
- Can you describe a moment when you were bored?
- Can you describe a moment when you felt alone?

Physical Health

- Are you able to run? Do you feel you can do physical activities other children can do? If no, can you describe these activities?
- Healthy children can do plenty of things in comparison to sick people, what are things you can do that sick children cannot do?

For young ones, ask the following:

- Are you sick sometimes? If you think of days when you are sick or you have pain, what do you do? What can't you do?

Psychological health/well-being

- Do you feel loved and cared? What makes you say that?
- Are you happy? Think about times you felt happy, or you laughed, can you talk about that time?
- On the other hand, what makes you sad? Tell us about the last time you cried. Is there someone you can talk to when you are sad?
- What makes you angry? How often are you angry?
- Do you feel appreciated? If your mom, teacher or older brothers and sisters were to tell us one of your qualities what would he or she say?
- Do you like yourself? what do you like about yourself? – use a mirror

- Do you look forward to growing up? Why?
- Now that you are almost an adult, how do you see your future? Do you worry? Are you optimistic? What are your plans?
- Are you satisfied about your life? Life goals?
- Do you feel you can make your own decisions? That you have control over your life. Do you get to relax and enjoy yourself? When and how do you do this?
- What do you want to do when you grow up? What do you dream of?
- What is it that you like most about your life? Is there one thing you would not change? Is there one thing you would like to change

How would you rate your quality of life? Can you explain (only for older group) –

Distribute a post-it to each participant and ask them to rate their QoL from 0 to 10 (0 being the worst and 10 being the best). Then each gets to explain their answer.
