# Supplementary material for: The development of the ScoPeO-Kids: A new measure of quality of life for children living in contexts of vulnerability
Source: PLOS Glob Public Health. 2026 Sep 9;6(9):e0007247. doi: 10.1371/journal.pgph.0007247 (PMC13557343; doi:10.1371/journal.pgph.0007247)
Supplement: Supplementary file 2 [file pgph.0007247.s004.pdf]

SCOPEO-KIDS QUESTIONNAIRE

To the interviewer:

Please use the information from the socio-demographic sheet to adapt the wording for the following concepts:

- If child does not live with parents, replace the word ‘parents’ by the person who is taking care of the child (e.g. grand-parent, other family member, tutor, guardian).
- When ‘home’ is mentioned, it refers to where the child is living now.
- If child doesn’t go to school, select the word that is adapted to the child (e.g. work, home, activities) when ‘school’ is mentioned in the question.
- Give the child the response scale page (page 2) so he/she can point his/her answer for each question.
- Mark the child’s answer for each question by putting an x in the appropriate column.

To the interviewer:

Please present the answers categories and related visual supports to the child.

For each question, please pick the word (or the circle) that best describes what you think or how you feel in general. Not necessarily today or yesterday but in your life in general.

Never	Not often/ a little	Often/ a lot	Always
○	○	○	○
**1**	**2**	**3**	**4**

In general would you say …		Never	Not often/ a little	Often/ a lot	Always	Did not answer
**Physical well-being dimension**						
1	… you are healthy					
2	… you can do activities other kids your age can do					
3	… you have enough food to eat					
**Emotional wellbeing dimension**						
4	… you are happy with your life					
5	… you feel loved					
6	… you feel you will be able to reach your goals/dreams when you are grown-up (*like earn money, continue schooling, have a family*)					
7	… you are worried					
8	… you are sad					
**Safety dimension**						
9	… you are afraid to go outside your house					
10	… there is fighting/quarrelling at home					
**Autonomy and self-realization dimension**						
11	… you have a chance to learn and develop new things/skills					
12	… you have time to do things you like					
13	… your parents are proud of you					
14	… your parents listen to your ideas/suggestions/opinions/what you think					
15	… you feel good about doing chores/housework at home (*dish washing, cooking, fetching water, collecting firewood* or other examples that are relevant for gender and country)					
**Social dimension**						
16	… you are good at making friends					
17	… you eat meals together as a family					

18	… you have someone to talk to about your feelings or worries/concerns					
19	… you participate in activities in your community (*going to church, wedding, sport day or other examples relevant for gender and country)*					
20	… Other kids are kind to you					

Thank you!
