# Supplementary material for: The development of the ScoPeO-Kids: A new measure of quality of life for children living in contexts of vulnerability
Source: PLOS Glob Public Health. 2026 Sep 9;6(9):e0007247. doi: 10.1371/journal.pgph.0007247 (PMC13557343; doi:10.1371/journal.pgph.0007247)
Supplement: Supplementary file 3 [file pgph.0007247.s006.pdf]

Dear Editor and Reviewers,

We thank you for your thorough review and constructive feedback on our manuscript entitled *“The development of the ScoPeO-Kids: A new measure of quality of life for children living in contexts of vulnerability.”* We address each suggestion in detail below (in blue). Line numbers refer to the updated version of the manuscript, which contains all the modifications. We have made significant revisions to the manuscript to address all points raised by reviewers.

Reviewer #1 Comments and Responses

Abstract

1. Reword the sentence on lines 34–35. “The pool of items…” to “The instrument was administered to….” This sentence was revised as suggested. Line 33.

2. Consider removing lines 37–41. These lines have been removed as suggested.

Introduction

3. Slow down the first paragraph; provide context before predictors. Take time to set it up for the reader. We have restructured the entire introduction paragraph to introduce the concept of quality of life before discussing predictors. Lines 43-102.

4. Line 94 – change “to the focus groups” to “of the focus groups.” Corrected as suggested. Line 107.

5. Line 97 – change “data was” to “data were.” Corrected as suggested. Line 110.

6. Lines 119–122 – briefly proved overview of frameworks and their relevance to your project. As suggested, we added a concise overview of the frameworks and how they were used in this study. Lines 132-150.

Method

7. Line 140 – provide a definition of slums and communities with a high prevalence of poverty. We added definitions with references to clarify these terms. Lines 170-174.

8. Move participant details from Results to Methods. We relocated participant characteristics in the Methods section. Line 202.

9. Line 154 – provide a rationale for use of these age groups. We added a rationale for age groups and included a description of protocol differentiation by age and provided a supplemental material as an Appendix. Lines 186-188.

10. Begin new paragraph at line 184. We adjusted paragraph structure accordingly. Line 228.

11. Provide details regarding thematic analysis (coders, process). We added number of coders, coding procedure, and consensus approach. Lines 232-234.

12. Lines 197-201 - How was demographic info provided (pencil and paper or tablet/computer?) collection and assistance for younger participants. We included details on data collection tools and support provided. Lines 242-243, 247-248.

13. Lines 222-223 - Provide more information of the 25 children who participated in the cognitive debriefing exercise (e.g. how many from each country, ages, genders). (country, age, gender). We expanded description along with requested details. Lines 277-293.

Results

14. Consider providing a table outlining key characteristics of QoL measures identified in the literature review. We appreciate the reviewer's insightful recommendation. Although a table summarizing current QoL measures might be helpful in the context of a thorough literature review, the introduction of this manuscript does not aim to provide a comprehensive overview of all available instruments. Rather, it aims to draw attention to conceptual and measurement gaps that facilitate the development of a new QoL measure. As a result, we chose to adhere to a brief narrative approach that focused on demonstrating the importance of the current study.

15. Workshop with HI should be moved to the Method section. Further details should be provided regarding workshop activities. We have addressed this comment as suggested. Lines 175-181

16. Line 247 – different word for "reuniting.". We have revised the wording by replacing 'reuniting by 'comprising'. Line 196.

17. Consider defining abbreviations in Table 2. We added the definitions below Table 2 (now Table 1). Line 203.

18. Line 270-271 – revise to read "Finally, parents associated the safety of the….". Sentence was rewritten as suggested. Line 326.

19. Table 3 – Additional details are needed. Provide some context for participants who said each of the quotes. Context was provided for each quote in Table 3.

20. Line 280 – Who compared findings from the workshop? How? We added explanation. Line 303.

21. Lines 291- 293 – Define “younger age” and “low reading skills.” Definitions and criteria were included as suggested. Lines 349-356.

22. Table 5 – Label the right column “n (%)” and remove “no. (%)” from each row. We revised as suggested.

23. Line 307 – More information is needed on the first factor (eigenvalue 9.6). Which items loaded on this factor? We revised the entire analyses section to meet with the suggestions of both reviewers. The original PCA analysis was removed and replaced by a PCA of the residuals as suggested.

24. Clarification is needed for lines 323 – 326. This is confusing as written. Sentences rewritten for clarity. Lines 409-412.

25. Figure 1 seems to be missing. We included Figure 1 on the submission portal as per instructions.

Discussion

26. The instrument was only tested with participants from 2 countries. Consider adding this as another limitation. This was added as a limitation as the end of the discussion section. Lines 537-540.

27. Lines 446-448 – Present this earlier in the Discussion, before the final paragraph. The final paragraph should be reserved for a brief overview and/or next steps. We repositioned as suggested. Lines 494-498.

Reviewer #2 Comments and Responses

Abstract

1. Line 40 – correct the grammar of the sentence "This analysis combined with cognitive debriefing sessions with led to the deletion of 23 items. " This sentence was deleted as suggested by first reviewer.

2. Line 48: The impacts – without s. We corrected grammar in line 40 and removed unnecessary “s” in “impacts.” Line 44.

Method

3. Line 100 - participants were recruited in 2017, like 8 years ago. What is the timing of this study, data collection etc. Does the setting of 2017 still apply today? A lot has changed with the new technologies and maybe also as consequence to the covid pandemia (I am thinking of life in refugee camps). We recognize that the data were collected 8 years ago. We acknowledge that the data were collected eight years ago. However, we have found recent studies describing living conditions in the slums of Dhaka and in refugee camps indicating that these contexts remain largely unchanged. In Dhaka, many households continue to experience moderate to severe socioeconomic and health vulnerability, characterized by insecure housing, severe overcrowding, and inadequate water, sanitation, and hygiene facilities. Similarly, in Thailand, refugees still live in semi-permanent camps, where freedom of movement is restricted and daily life remains heavily dependent on humanitarian assistance. Moreover, this questionnaire is designed for children living in contexts of vulnerability and we believe that the contexts in which data were collected is representative of other regions in the world today.

Bibliography: Farzana, F. D., Hossain, D., Rahman, M., Nahar, Q., Arifeen, S. E., Ahmed, T., & Mahfuz, M. (2024). *Understanding water, sanitation, and hygiene situation through participatory appraisal in urban slum of Dhaka City*. European Journal of Environment and Public Health, 8(4), em0165. https://doi.org/10.29333/ejeph/15585

Hasan, M. Z., Rabbani, M. G., Ahmed, M. W., Mehdi, G. G., Islam, K. I. T., Reidpath, D. D., … Mahmood, S. S. (2024). Assessment of socioeconomic and health vulnerability among urban slum dwellers in Bangladesh. *BMC Public Health*. https://doi.org/10.1186/s12889-024-20425-9

UNHCR. (n.d.). *Thailand*. Retrieved January 2026, from https://www.unhcr.org/where-we-work/countries/thailand

Intesar, A. (2024). Living with vulnerability: Triple burden through the eyes of urban slum women in Bangladesh. Social Sciences & Humanities Open, 10, Article 101014.

Hou, T. (2024). Humanitarian aid practices on the Thai-Myanmar border after the 2021 coup. Journal of Humanitarian Affairs, 00159

4. Lines 119-122 – Please rephrase this sentence: The models proposed by Round and colleagues (13) and Fagerlind schematisation, completed by Maslow’s goals or needs perspective (14), were used as frameworks to support the reflexion behind the development of the ScoPeO-kids. Ex. – Several frameworks

supported the reflexion … We rephrased sentence on frameworks for clarity. Lines 143-147.

5. Line 149: pooled by domains? We have rewritten this sentence. Lines 176-181.

6. Line 200: Bangladesh. Corrected throughout the text.

Rasch Analysis and PCA:

7. Line 207: I am unsure if the PCA was done on the data or on the residuals of the Rasch analysis. It reads like the PCA was done before the Rasch analysis in SAS. I don't think that that PCA is valid approach on the raw, ordered data, or maybe with polychoric correlations or strategy to have interval scaled data. In RUMM2030, PCA of residuals can be tested to decide if a set of items is unidimensional. I would rather suggest making the test of dimensionality, and also the selection of items in RUMM2030 - and check the eigenvalues and component loadings there instead of a separate analysis. We have modified this entire section according to the suggestion of reviewer and specified that PCA of residuals was conducted within Rasch framework using RUMM2030. We added details on interpretation and rephrased sentences on fit statistics and monotonicity. We have clarified threshold ordering and DIF analysis. We have also added an explanation of reliability assessment using the Person Separation Index (PSI) and its interpretation. Lines 250-275.

8. Line 209: Fit was assessed to verify fit… Please rephrase following sentence "Overall fit and individual item fit were assessed to verify adequate fit of the items and their response options to the model." The item fit does not say anything about the threshold ordering. We rephrased accordingly – see lines 250-275.

9. Line 212 – "Rasch analyses were used to detect biases "– suggestion to rather write for example "An analysis of differential item functioning allowed to investigate lack of invariance within items for groups of age and country of origin. We rephrased as suggested. Lines 269-271.

10. Line 214: "Monotonocity was also verified through fit statistics" – Monotonicity is not verified with the fit statistics. You could find disordered thresholds in items with good fit as well as, reversely, misfitting items with ordered thresholds. Monotonicity is investigated by means of the estimates of the response difficulty thresholds and their ordering. This sentence was rephrased. Line 261.

11. Line 215 – here (and in this entire section), the information could be more specific. Reliability is assessed with the person separation index, where values above 0.85 are considered good fit and above 0.7 as sufficient… or is interpreted similarly to Cronbach alpha… Later the authors write about person separation, so it would make sense to mention it in the method section already. We have

rewritten this section to improve clarity and have added reliability in the Methods section. Lines 270-272.

12. Line 215 could the authors say more about the response options for the selected questions already here? We have included details on response options as indicated. Lines 306-308.

13. Line 288 – the information about the response options could be provided earlier. We moved it up at the beginning of the Results section. Lines 308-310.

14. Line 306 – it is quite uncommon to use the Kaiser criterium (1st eigen <1) to decide on the dimensionality of a scale - when performin PCA on residuals... Based on Linacre 1st eigenvalues > 2 are strong indication for multidimensionality or Smith and Miao suggest a second eigenvalue > 1.4. As mentioned above, this section was rewritten. A PCA of the residuals is now presented.

15. Line 225 – were the children interviewed? It surprises me that children of age 5 are already completing a questionnaire. How were the interviews done for the children that could not read? All children, including the youngest participants, were interviewed using an interviewer-administered cognitive debriefing approach. Trained interviewers were able to adapt their language to the child's age and used visual aids when necessary.

16. Line 304 – what does IDP stand for? (make sure that all acronyms can be understood). We have defined this acronym (Internally Displace Persons). Line 363.

Results

17. Line 310 – With a a PCA, you would have component loadings (and not factors) and eigenvalues. You would have as many components as items in the scale. You would first observe which items load positively or negatively on the first component. You could also plot the values of the first against the second component and see which items cluster etc…We have reorganized confusing sections on Rasch model requirements and fit and have presented the results of the PCA of the residuals. Lines 373-482.

18. Line 313. "Analysis of the 43 items indicated that not all items met the requirements of the Rasch model". This section is confusing as it first mentions the items (that do not meet requirements of the Rasch model). Than, it is about the model fit. Also requirements of the Rasch model are not only about the item fit – threshold ordering, local independence and absence of DIF are also important requirements. We have reorganized confusing sections on Rasch model requirements and fit. Lines 366-416.

Line 317 – please round it e.g. p-value < 0.001. We rounded p-values appropriately. Line 371.

19. Line 319 – the PSI is very likely to be inflated - because of remaining dependencies and multidimensionality. We have added a sentence to this effect in the discussion. Lines 506-509.

20. Line 323 - Please explain what it means to have a big score versus a small score on the scale. We have explained meaning of high vs. low scores on the scale. Lines 346-347.

21. In this section, I miss an analysis of local item dependencies (LID). It would be interesting to know how the multidimensionality and LID are connected, if the selection of items show groups of items. Absence of local item dependencies would provide unbiased estimate of reliability. We added analysis of LID. Lines 262-264 and 400-402.

22. Line 364 – A response scale that mixes perspectives can be confusing and may be a limitation. Your response scale suggests as option not often/a little and often/ a lot. (I am not sure about the phrasing of the items but - "things" can happen often but with little effect also and reversely can happen rarely but with big effect. In that sense, a unidimensional response mode would be less ambiguous (intensity even better than frequency). For example, items on conflicts at home - if seldom but when, than very violent - what response is it not often or a lot? Thank you for this comment. The response format was designed to capture participants' *overall perceived burden* of each experience rather than to disentangle frequency and intensity as separate constructs. During the development phase, cognitive interviews indicated that respondents tended to integrate how often an experience occurred and how impactful it was into a single global judgment, which informed our choice. We acknowledge that future research may require exploration of alternative response formats.

Discussion

23. The discussion is interesting, but many paragraphs are too closely describing results that could have been or were mentioned previously. In that sense the discussion section needs some work in deciding what is a discussion point or a result. Also this discussion could benefit of more information regarding the implication of this study, the advantage of this tool versus existing tools. We restructured and rewrote this section. We reduced repetition of results; built on implications and advantages of ScoPeO-Kids.

24. Line 446 – Regarding the conclusion – the authors mention that the ScoPeO-kids tool is needed because two domains are specific to children in slums or low socio-economic communities (safety and autonomy/self-realization). How are

these items specific to this population? Are they not important for all children? Do other tools omit to assess these aspects and for this reason the ScoPeO-kid is a plus? We agree that safety and autonomy are fundamental aspects of quality of life for all children. Our intention was not to suggest that these domains are exclusive to children living in contexts of vulnerability, but rather that their contribution to QoL differ substantially in settings such as refugee camps or slums. In our study, these dimensions emerged as distinct domains. We expanded the discussion on domains specific to vulnerable contexts and why they add value beyond existing measures. We have added that commonly used pediatric QoL instruments often assess safety and autonomy implicitly or indirectly. Lines 498-502.

Additional Changes

- We corrected the grammar and improved clarity throughout.

- We updated references.

We believe these revisions significantly strengthen the manuscript and address all concerns raised. Thank you again for your valuable feedback.

Kind regards,

Johanne Higgins, PhD
Professeure titulaire|Full professor
École de réadaptation
Pavillon du Parc, Bureau 403-18
C.P.6128 Succ. Centre-ville,
Montréal QC, Canada, H3C 3J7
Adresse civique: 7077 av. du Parc
Montréal, QC
H3N 1X7

Chercheuse |Researcher, Centre de recherche interdisciplinaire en réadaptation du Montréal métropolitain (CRIR)
Site de l’Institut universitaire sur la réadaptation en déficience physique du CIUSSS Centre-Sud-de-l'Île-de-Montréal.
