# Supplementary material for: The development of the ScoPeO-Kids: A new measure of quality of life for children living in contexts of vulnerability
Source: PLOS Glob Public Health. 2026 Sep 9;6(9):e0007247. doi: 10.1371/journal.pgph.0007247 (PMC13557343; doi:10.1371/journal.pgph.0007247)
Supplement: Supplementary file 4 [file pgph.0007247.s007.pdf]

Université de Montréal
Faculté de médecine
École de réadaptation

CRIR
Centre de recherche interdisciplinaire en réadaptation du Montréal métropolitain

Montreal April 6, 2026

Dear Editor and Reviewers,

Thank you for your thorough and helpful review, which made the paper more rigorous overall. We have responded to each comment in detail, point by point, below. All changes have been made to the new version of the manuscript, and they are clearly indicated using track changes.

Responses to Reviewer #3

1. Clarification of theoretical framework (Maslow and others)

Comment: Although the introduction provides a strong rationale for the study, it could better articulate the theoretical frameworks that inspired the study. Frameworks such as Maslow's hierarchy are mentioned, but their specific application to the development of this measure is not clear. How did Maslow's theory of needs help shape the selection of domains such as safety or social well-being? A clearer link of theory to the development of the items would strengthen the conceptual foundations.

Response: We added a section in the introduction (p. 5; lines 96-101):

*"*The conceptual frameworks shaping ScoPeO-Kids help identify and categorize domains and do not impose a strict structure. Domains concerning physical health, autonomy, and emotional well-being are informed by frameworks that emphasize functioning, capabilities and subjective well-being. Furthermore, Maslow's hierarchy of needs is used to prioritize basic needs and safety as fundamental components of QoL in vulnerable groups.*"*

We have also added a paragraph in the Methods section (p. 9; lines 178-186).

"Drawing on Maslow's hierarchy, the measure was developed on the premise that QoL among vulnerable children is closely linked to the satisfaction of basic needs. Basic needs such as food, shelter and security were also put forward by field experts from HI and the literature as major concerns to be addressed when working with children living in refugee camps and slums (20), directly informing their inclusion. Social and emotional well-being domains were also considered to reflect higher-level needs, conceptualized as dependent on, and interacting with, the fulfilment of basic needs.

We have included a paragraph in the discussion (p. 30; lines 596-600): ***"****Maslow's hierarchy showed how basic needs, like having enough food and feeling safe, affect how children living in vulnerable situations think about their QoL. The ScoPeO-Kids domain structure combines established QoL frameworks with the real-life experiences of vulnerable children, strengthening its conceptual basis."*

2. Item selection and exclusion process

Comment: The rationale for excluding specific items (e.g., "balance between play/leisure and work") and the decision-making process for prioritizing items are not fully explained in the introduction. This raises

questions about the subjective nature of item selection. Therefore, explain how the input from different stakeholders (e.g., parents, children, professionals) was weighed and balanced.

Response: We appreciate this comment and have added a paragraph in the Introduction section (p. 5–6; lines 102-112) to clarify: "*Because QoL is multidimensional and specific to each context, the development of ScoPeO-Kids was based on a structured process aimed to reduce subjectivity in the selection of domains and items where the opinions of stakeholders—children, parents, and field professionals—were synthesized in a complementary way so as to achieve a balance among conceptual relevance, as well as ecological validity. Items were selected based on their consistency across groups, cultural relevance, and direct connection to children's lived experiences. In contrast, ideas commonly found in QoL measures but not as relevant in humanitarian settings were not included. This approach ensured that the items chosen were based on shared priorities and not random or purely theoretical ones.*"

3. Clarity of Rasch analysis explanation

Comment: While a Rasch analysis is performed to confirm the psychometric properties of the instrument, the results section lacks a comprehensive explanation of the statistical method. For example, the explanation of "item-attribute interaction" and "fit residuals" is too technical for readers who may not be familiar with Rasch analysis.

Response: We have added brief explanations of Rasch concepts in the Results section. (Pages 23-26).

4. Inclusion/exclusion criteria and representation of children with disabilities

Comment: In the methodology section, provide more information about the inclusion/exclusion criteria for participants, particularly with regard to children with disabilities. Clarify whether specific steps were taken to ensure adequate representation of the diversity within this group (e.g., different levels of disability severity).

Response:
Thank you for this comment. We have clarified the inclusion and exclusion criteria for participants, including children with disabilities. This paragraph was added (p. 11-12; lines 229-240).

"Children and parents were identified and recruited through HI's local field teams and partner organisations operating in the selected refugee camps, slums and host communities. Potential participants were approached through existing community structures, such as schools, community centers and program activities. Field staff were instructed to recruit children who were broadly representative of the local population served, including children with and without disabilities, and across age groups, and to ensure the inclusion of children with diverse functional profiles to reflect the heterogeneity of the population served by HI. Children with disabilities were eligible to participate provided they had the cognitive capacity and willingness to engage in group discussions and could communicate verbally. No exclusion criteria were applied based on the type or severity of disability."

Another paragraph was added (p. 15, lines 310-314): "Children with disabilities were included in the survey phase following the same criteria as other children. The recruitment process aimed to reflect the

diversity of children receiving services, including those with different functional limitations. To help with participation, interviewer-led interviews and visual aids were used as needed."

We have also acknowledged this in the discussion: "Also, participant recruitment through partner organisations may have introduced selection bias, as children not engaged in community services or programs could have been less likely to be included."

5. Suggested references

Comment: Suggested inclusion of two articles to strengthen contextualization. I would like to kindly suggest citing the following articles, which I believe could provide additional context and reinforce certain points within your manuscript:
Article 1: Kazemi Karyani A, Rezaei S, Karami Matin B, Amini S. Poor health-related quality of life in Iran: decomposition analysis of affecting factors. International Journal of Human Rights in Healthcare. 2019 Feb 21;12(1):28-37.
Comment: "The quality of life (QoL) for individuals in vulnerable populations can be significantly impacted by factors such as poverty, disability, and environmental conditions, making it essential to consider these contextual elements when designing and evaluating QoL measures."
Placement in Manuscript: This comment can be inserted after the sentence ending with "need for a tool capable of measuring the outcomes of such interventions on the QoL of children regardless of their gender, age or disability status."
Article 2: Nazari J, Jafari K, Chegini M, Maleki A, MirShafiei P, Alimohammadi A, Kazemzadeh Y, Mikaeliyan R, Amini S. Physical and mental growth and development in children with congenital hypothyroidism: a case–control study. Orphanet journal of rare diseases. 2021 Sep 23;16(1):393.
Comment: "Children's physical and mental growth, as well as their emotional well-being, are deeply interconnected and influenced by a range of social, environmental, and psychological factors that should be assessed holistically in any quality of life measure. Placement in Manuscript: This comment can be inserted after the sentence ending with "understanding the factors that influence the QoL in vulnerable children is of utmost importance to deliver evidence-based interventions."

Response: We thank the reviewer for these helpful suggestions. Both references have been incorporated into the Introduction (pp. 5 and 7; lines 92-96 and 128-131) at the recommended locations.

Response to Reviewer #4

1. Conceptual framework coherence

Comment: The manuscript references several conceptual perspectives on quality of life, yet it is not entirely clear how these frameworks informed the development of the final domains. More explicit explanation of the relationship between the initial conceptual framework and the final five-domain structure would strengthen the interpretability as well as coherence.

Response: We thank the reviewer for this comment. We have clarified how the initial conceptual frameworks informed the development of the final structure. Specifically, the development of the ScoPeO-Kids was guided by complementary conceptual perspectives on QoL, including functioning,

capabilities and subjective well-being frameworks, as well as Maslow's hierarchy of needs. These perspectives informed the identification and prioritization of domains, while the final domain structure emerged through an iterative process integrating empirical evidence from children, parents and field professionals, and psychometric refinement using Rasch analysis. See answer to reviewer 3 – comment 1.

2. Definition of vulnerability

Comment: How the authors defined 'vulnerability' should be made clearer. A clearer definition of the target population would help readers understand the instrument's contexts of applicability.

Response: We have improved the definition of vulnerability by adding a clear definition in the Introduction (p. 4; lines 72-77): "*In this study, vulnerability is defined as a situation of significant exposure to threats or hazards that may limit the satisfaction of basic needs and access to fundamental rights, depending on individuals' capacity to anticipate, cope with, or recover from these threats. Vulnerable children therefore include those living in poverty, social exclusion, conflict, displacement or disaster, and who may also present with a disability.*"

3. Sampling and recruitment procedures

Comment: More detail regarding participant recruitment would improve transparency, e.g. how children and caregivers were identified and recruited through partner organisations, and whether there might have been selection bias.

Response: We have added further details to the Methodology section to clarify participant recruitment procedures. See answer to reviewer 3, comment 4 above.

4. Psychometric reporting and ceiling effect

Comment: It would also be helpful to include key item statistics (e.g., item difficulty and fit indices) in a supplementary table. Additionally, the implications of the observed ceiling effect should be discussed in greater detail, notably regarding the instrument's sensitivity to detect change in intervention evaluations.

Response: A **supplementary table** has been added as supplementary material presenting item-level statistics (difficulty, fit indices).

The Discussion (pp. 34; lines 635-642) now includes an expanded interpretation of the observed ceiling effect. *"The targeting analyses indicated a slight ceiling effect, with some children presenting levels of QoL higher than those targeted by the most 'difficult' items. This suggests that, in subgroups of children with relatively higher baseline QoL, the instrument may have reduced sensitivity to detect small improvements over time. Given that the ScoPeO-Kids was designed for use in settings where the baseline QoL is often poor, the ceiling effect is not likely to significantly limit its ability to respond in intervention evaluations for children with low to moderate QoL."*

5. Cross-cultural validity and translation

Comment: The instrument was administered across several countries and languages, yet the author provides limited detail on the translation and cultural adaptation procedures, which could be important for increasing confidence in the instrument's applicability.

Response:
Thank you for this comment. The manuscript has been revised. This was added p.14-15, lines 297-309. *"The questionnaire was initially developed in English and subsequently translated into the relevant local languages (Karen and Burmese in Thailand; Bangla in Bangladesh; Urdu in Pakistan). Translation was coordinated and supervised by HI's local field teams in each country, who have extensive experience working with children in humanitarian and low-resource settings. While a formal forward–backward translation procedure was not systematically documented, particular attention was paid to cultural and contextual appropriateness of item wording. To support cross-language equivalence, cognitive debriefing interviews were conducted with children in each country to assess item comprehension, relevance and interpretation. Findings from the cognitive debriefing indicated that children across languages interpreted the items as intended, suggesting that the translations did not introduce systematic interpretation bias".*

We have also acknowledged this as a methodological consideration in the Discussion. (p. 34; lines 669-673): "Finally, translations of the questionnaire were supervised by experienced field teams and supported by cognitive debriefing. However, the absence of a formally documented forward–backward translation process represents a limitation; future studies could further strengthen cross-cultural equivalence by incorporating standardized translation protocols."

6. Operational use and interpretation of scores

Comment: The discussion could also further explain how the instrument should be used operationally, including how scores should be interpreted in program evaluation contexts.

Thank you for this suggestion. We have expanded the Discussion to clarify how the ScoPeO-Kids is intended to be used. We explain how scores should be interpreted at the group level, how they can inform monitoring of change over time. (p. 34; lines 676-685)

We thank the reviewers for their comments, which we believe have significantly improved the clarity and rigor of our manuscript. We hope that the revisions satisfactorily address all concerns raised by the reviewers.

Sincerely,
Johanne Higgins
On behalf of all co-authors
