# Supplementary material for: The development of the ScoPeO-Kids: A new measure of quality of life for children living in contexts of vulnerability
Source: PLOS Glob Public Health. 2026 Sep 9;6(9):e0007247. doi: 10.1371/journal.pgph.0007247 (PMC13557343; doi:10.1371/journal.pgph.0007247)
Supplement: Supplementary file 5 [file pgph.0007247.s001.docx]

|  | | | | | **Chi-Square** | | **F-stat** | |
| --- | --- | --- | --- | --- | --- | --- | --- | --- |
| **Items** | | **Location** | **Standard Error** | **Fit Residual** | **Value** | **p-value** | **Value** | **p-value** |
| **1** | In general, I feel healthy | -0.076 | 0.049 | 3.551 | 11.526 | 0.241 | 1.020 | 0.422 |
| 2 | In general, I consider myself a happy person | -0.143 | 0.053 | -0.249 | 10.583 | 0.305 | 1.195 | 0.295 |
| 3 | I feel that I live in a loving home | -0.236 | 0.053 | 0.477 | 10.014 | 0.349 | 1.112 | 0.352 |
| 4 | I fell god about doing chores at home | 0.022 | 0.051 | 0.413 | 19.796 | 0.019 | 1.801 | 0.065 |
| **5*** | I feel good about needing help to feed, dress or bathe myself | 0.387 | 0.041 | 7.554 | 49.283 | 0.000 | 3.702 | 0.000 |
| 6 | I can play games outside or do sports that other kids my age can do | -1.633 | 0.051 | 0.749 | 6.134 | 0.726 | 0.626 | 0.776 |
| 7 | I have fun with my friends | -0.169 | 0.054 | 0.366 | 12.812 | 0.171 | 1.360 | 0.203 |
| **8** | I have playgrounds/places outside where I can play safely | 0.537 | 0.041 | 2.87 | 17.324 | 0.044 | 1.400 | 0.185 |
| 9 | I am happy with playgrounds/places outside where I can play | 0.366 | 0.041 | 2.115 | 7.384 | 0.597 | 0.692 | 0.716 |
| 10 | I feel fit and healthy | -0.030 | 0.049 | -0.836 | 24.061 | 0.004 | 2.917 | 0.002 |
| 11 | I can eat, dress and wash by myself | -0.336 | 0.052 | 1.036 | 14.099 | 0.119 | 1.395 | 0.187 |
| 12 | I can eat, dress and wash by myself | -0.041 | 0.049 | 0.404 | 19.550 | 0.021 | 2.274 | 0.016 |
| **13*** | I can have fun even when I have pain | 0.896 | 0.039 | 7.240 | 101.056 | 0.000 | 8.196 | 0.000 |
| 14 | I have enough food to eat everyday | 0.025 | 0.048 | 0.091 | 6.393 | 0.700 | 0.707 | 0.703 |
| 15 | I have someone who listens to me when I need to talk | 0.110 | 0.051 | -0.323 | 14.897 | 0.094 | 1.626 | 0.105 |
| 16 | My father/mother listens to my ideas | -0.099 | 0.053 | -2.350 | 24.411 | 0.004 | 3.309 | 0.001 |
| 17 | My parents allow me to do most of the activities I want | 0.160 | 0.048 | 1.459 | 16.812 | 0.052 | 1.761 | 0.073 |
| **18** | I feel I can make my own decisions | 0.695 | 0.043 | 4.582 | 28.566 | 0.001 | 2.678 | 0.005 |
| 19 | I have opportunities for learning skills and knowledge that I feel I need | 0.175 | 0.047 | -1.508 | 14.984 | 0.091 | 1.799 | 0.066 |
| **20** | I don’t feel different from other children | 0.331 | 0.045 | 3.855 | 14.222 | 0.115 | 1.233 | 0.272 |
| 21 | Other kids are nice to me | -0.106 | 0.053 | 0.083 | 4.157 | 0.901 | 0.479 | 0.889 |
| **22*** | I can play or spend time with my friends even when I have pain | 0.792 | 0.041 | 5.942 | 49.977 | 0.000 | 4.240 | 0.000 |
| **23*** | I am happy with my life | -0.302 | 0.056 | -2.539 | 30.56 | 0.000 | 4.426 | 0.000 |
| **24*** | I do not want to change anything in my life | 0.668 | 0.038 | 7.347 | 62.529 | 0.000 | 4.331 | 0.000 |
| **25*** | I rarely cry | 0.695 | 0.043 | 8.156 | 138.032 | 0.000 | 11.111 | 0.000 |
| **26*** | I rarely feel sad | 0.440 | 0.048 | 5.608 | 94.891 | 0.000 | 8.275 | 0.000 |
| 27 | I feel safe when I’m at home | -0.080 | 0.053 | -0.897 | 22.078 | 0.009 | 2.661 | 0.005 |
| 28 | I feel loved | -0.349 | 0.059 | 0.269 | 10.106 | 0.342 | 1.061 | 0.390 |
| 29 | There are adults who encourage me to do my best | -0.628 | 0.058 | -1.127 | 12.949 | 0.165 | 1.55 | 0.127 |
| 30 | I feel I will be able to reach my goals/dreams | 0.060 | 0.051 | -2.002 | 20.892 | 0.013 | 2.697 | 0.004 |
| 31 | I feel hopeful about my future | -0.433 | 0.056 | -1.584 | 21.276 | 0.011 | 2.685 | 0.005 |
| 32 | I like myself | -0.299 | 0.055 | 0.592 | 16.613 | 0.055 | 1.805 | 0.064 |
| 33 | I can play as much as I want in my free time | -0.001 | 0.050 | -0.908 | 14.127 | 0.119 | 1.65 | 0.098 |
| 34 | I can use my free time as I want to | 0.039 | 0.051 | -0.481 | 14.099 | 0.119 | 1.616 | 0.107 |
| 35 | My parents spend time with me doing things that I like | 0.065 | 0.054 | -0.78 | 17.241 | 0.045 | 1.958 | 0.042 |
| 36 | I get along well with my friends | -0.081 | 0.055 | -0.299 | 14.262 | 0.113 | 1.560 | 0.124 |
| 37* | I feel accepted by other kids | -0.150 | 0.057 | -2.422 | 48.372 | 0.000 | 7.117 | 0.000 |
| 38 | I can talk to my parents when I want to | -0.277 | 0.059 | -1.055 | 13.338 | 0.148 | 1.596 | 0.113 |
| 39* | I am able to talk about everything with my friends | -0.143 | 0.054 | -2.132 | 31.855 | 0.000 | 4.289 | 0.000 |
| 40* | I am good at making friends | -0.314 | 0.058 | 0.127 | 31.237 | 0.000 | 3.613 | 0.000 |
| 41* | I spend time with my friends as I want to | -0.279 | 0.053 | -1.332 | 33.589 | 0.000 | 4.274 | 0.000 |
| 42 | I feel my father/mother are proud of me | 0.089 | 0.049 | 0.943 | 13.526 | 0.140 | 1.370 | 0.199 |
| 43* | I eat meals with my family as often as I | -0.348 | 0.058 | -1.391 | 43.347 | 0.000 | 6.015 | 0.000 |

Positive location (measure) numbers mean harder items; negative location means easier items.

Bolded items: outside the ±2.5 range for fit residuals.

*fit residuals with significant chi-square and\or F-statistics (p <0.000233).
