# Supplementary material for: The development of the ScoPeO-Kids: A new measure of quality of life for children living in contexts of vulnerability
Source: PLOS Glob Public Health. 2026 Sep 9;6(9):e0007247. doi: 10.1371/journal.pgph.0007247 (PMC13557343; doi:10.1371/journal.pgph.0007247)
Supplement: Supplementary file 6 [file pgph.0007247.s002.docx]

Inclusivity in global research

PLOS’ policy on inclusivity in global research aims to improve transparency in the reporting of research performed outside of researchers’ own country or community and ensures that PLOS publications reporting global research adhere to high standards for research ethics and authorship. Authors of relevant research articles may be asked to complete the questionnaire below, which outlines ethical, cultural, and scientific considerations specific to inclusivity in global research. This questionnaire may be requested when researchers have travelled to a different country to conduct research, if research uses samples collected in another country, research with Indigenous populations or their lands, or if research is on cultural artefacts. Researchers travelling to another country solely to use laboratory equipment will not normally be required to complete the questionnaire. However, the questionnaire can be requested at the journal’s discretion for any submission – if you have been requested to complete this questionnaire by the PLOS journal you submitted to, please do so.

Please complete the questionnaire below and include this as a Supporting Information file with your manuscript. Note that if your paper is accepted for publication, this checklist will be published with your article in the supporting information files. Please ensure that you reference the checklist in the main body of your manuscript. We suggest adding a subsection ‘Inclusivity in global research’ to your Methods section and adding the following sentence: “Additional information regarding the ethical, cultural, and scientific considerations specific to inclusivity in global research is included in the Supporting Information (SX Checklist)”

The questions have been designed to be applicable to a wide range of study types, and there are subsections for both human subjects research and non-human subjects research. If any of the questions are not relevant to your research please mark them as “N/A” as appropriate.

**Ethical considerations, permits and authorship**

*This section is applicable to all research types.*

Provide details as to who granted permissions and/or consent for the study to take place in the Methods section of your manuscript. This should include the names of **all** ethics boards, governmental organizations, community leaders or other bodies that provided approval for the study. If individuals provided approval refer to these people by their role or title but do not list their name(s).

Ethics reported on page number: page 5, lines 92-93. Also, authors would like to mention that in addition to receiving approval from the University of Montreal’s ethics committee, the research adhered to international ethical standards. It also complied with the internal standards of the partner non-governmental organization, which reaffirms the implementation of procedures to protect participants and research teams, ensure data security, guarantees the collection of informed consent from all participants - both children and parents, adapts the research methods to the specific needs of participants and respects socio-cultural norms. Finally, local authorities at community levels were informed and granted authorization for the implementation of the project.

If there were any deviations from the study protocol after approval was obtained please provide details of these changes in the Methods section of your manuscript.

No deviation from the study protocol to report.

Did this study involve local collaborators that are residents of the country where the research was conducted or members of the community studied? If you do not have any authors from said communities, please provide an explanation for this below.

Yes. The study involved local collaborators based in the country where the research was conducted. These collaborators supported study implementation and provided contextual expertise. The research mobilised local resources. Teams from the partner NGO were engaged locally, particularly to facilitate the implementation of the study by identifying communities & participants, introducing the project and research team to local authorities, supporting the translation of the questionnaire into the local language, participating in data collection to ensure that it was child-friendly and respectful of the cultural context. They were mainly involved in planning and fieldwork. They are not included as authors because they did not fulfill the journal’s authorship requirements.

Everyone listed as an author should meet PLOS’ criteria for authorship and all individuals who meet these criteria should be included in the author byline, rather than the acknowledgements. For further information please see the journal’s Authorship Policy.

**Human subjects research (e.g. health research, medical research, cross-cultural psychology)**

Did you obtain written informed consent from a representative of the local community or region before the research took place? How did you establish who speaks for the community? Details of written informed consent obtained from study participants should be reported separately in the Methods section of your manuscript.

Before the start of the study, HI staff performed groundwork to find people and groups in the area that were known as legitimate representatives of the community. These representatives were chosen based on the current governance, health, and community structures. Although formal written consent at the community level was not acquired, verbal agreement and authorization to proceed were obtained through these designated representatives. As stated in the Methods section, all participants gave their written consent to take part in the study.

How did members of the local community provide input on the aims of the research investigation, its methodology, and its anticipated outcome(s)?

Local community members did not provide direct input into the research aims, methodology, or anticipated outcomes. However, the study design was informed by contextual knowledge and operational experience provided by partner NGO personnel, based on their long-standing engagement with the communities. Indeed, the dimensions and items defining quality of life were not identified solely from a review of the literature and international and in country expert feedback. The methodology also incorporated the perspectives and feedback of children and parents through focus group discussions, also to ensure that the approach was grounded in the lived experiences of the target populations.

When engaging with the local community, how did you ensure that the informed consent documents and other materials could be understood by local stakeholders?

All informed consent documents and study materials were translated into the relevant local language(s) and adapted/simplified when necessary for younger children. Translations were reviewed by HI personnel and local staff familiar with the language as well as the culture. Study procedures were explained verbally to participants, and opportunities were provided to ask questions before consent was obtained.

Will the findings of the research be made available in an understandable format to stakeholders in the community where the study was conducted (e.g. via a presentation, summary report, copies of publications, etc.)? Please provide details of how this will be achieved.

Feedback to the main community stakeholders was not formalized, primarily due to delays in the delivery of the final approach.

It should be noted that this research was part of a broader four-year project aimed at promoting the right to play for refugee children in Bangladesh, Pakistan, and Thailand.

The research led to the development of an approach for measuring the impact of development projects, known as *ScoPeO-Kids*, as well as the production of a methodological guide co-created with the project teams. This approach and related guidance was shared with the project’s country teams and more broadly within the partner NGO, which has since systematized its use to assess the outcomes of its projects on children’s quality of life.

**Non-human subjects research using specimens/ animals collected as part of the study, or those housed in archival collections. Examples include archaeology, paleontology, botany and zoology.**

Did the permission you obtained from a local authority to perform the study include an agreement on access to outputs and benefit sharing? This may include procedures to enable fair distribution of the benefits and resources arising from the research performed. Please include any details of Prior Informed Consent and Benefit Sharing Agreements obtained. These may be required by field-specific regulations, for example the Convention on Biological Diversity (CBD) and the associated Nagoya Protocol.

N/A

If the material used in your study was imported, please A) provide the year it was imported and B) indicate whether permits were obtained to import/export the materials used, C) provide details of any permits obtained. If this information is not available, please indicate this.

N/A

If you used archival specimens, please state how the material used in your study was acquired by the institute it is held in and provide details of any permits obtained for the original excavations/ sample collection. If this information is not available, please indicate this.

N/A

How was the potential cultural significance of the materials collected in your study to local communities considered in your research design? Were Indigenous peoples and/or local researchers and institutions involved with archaeological excavations / collection of specimens? If so, please provide a description of their involvement.

N/A

If your manuscript includes photographs of human remains please indicate whether authors obtained permission from descendants or affiliated cultural communities to do so.

N/A
