# Supplementary material for: The development of the ScoPeO-Kids: A new measure of quality of life for children living in contexts of vulnerability
Source: PLOS Glob Public Health. 2026 Sep 9;6(9):e0007247. doi: 10.1371/journal.pgph.0007247 (PMC13557343; doi:10.1371/journal.pgph.0007247)
Supplement: Supplementary file 7 [file pgph.0007247.s005.docx]

Thank you for the opportunity to review the manuscript, “The development of the ScoPeO-Kids: A new measure of quality of life for children living in contexts of vulnerability.” The purpose of this project was to develop an instrument to measure quality of life for children living in situations of vulnerability. This was an interesting paper, but revisions must be made before the paper is suitable for publication. Most importantly, more details are needed throughout the Method and Results sections. Please see recommendations below:

**Abstract**

Reword the sentence on lines 34-35 beginning with “The pool of items…” to read “The instrument was administered to….”

Consider removing lines 37-41.

**Introduction**

Slow down a bit. The first paragraph jumps right into predictors of quality of life. Take some time to set this up for the reader.

Line 94 – change “to the focus groups” to “of the focus groups…”

Line 97 – data “were”

Lines 119-122 – take some time to briefly provide an overview of the frameworks and why they were particularly relevant to your project.

**Method**

Line 140 – provide a definition of slums and communities with a high prevalence of poverty

Provide details of participants in the Children Focus Groups and Parents Focus Groups sections (p. 8-9) rather than in the Results.

Children Focus Groups section

- Line 154 – provide a rationale for the use of these age groups
- Describe how the protocols were differentiated based on age. Provide protocols as a table or supplemental material.

Begin a new paragraph, starting with “All focus group discussions were audio-recorded….” (line 184).

Provide additional details regarding thematic analysis (lines 186-188). Example, how many coders analyzed data? What did the process look like.

Lines 197-201 – How was demographic info provided (pencil and paper or on a tablet or computer?). Describe any help provided to younger participants.

Lines 222-223 – Provide more information of the 25 children who participated in the cognitive debriefing exercise (e.g., how many from each country, ages, genders)

**Results**

Consider providing a table outlining key characteristics of QoL measures identified in the literature review.

Workshop with HI should be moved to the Method section. Further details should be provided regarding workshop activities.

Line 247 – different word for “reuniting.”

Consider defining abbreviations in Table 2.

Line 270-271 – revise to read “Finally, parents associated the safety of the….”

Table 3 – Additional details are needed. Provide some context for participants who said each of the quotes.

Line 280 – Who compared findings from the workshop? How?

Lines 291- 293 – Define “younger age” and “low reading skills.” How were reading skills determined?

Table 5 – Label the right column “n (%)” and remove “no. (%)” from each row.

Line 307 – More information is needed on the first factor (eigenvalue 9.6). Which items loaded on this factor?

Clarification is needed for lines 323 – 326. This is confusing as written.

Figure 1 seems to be missing.

**Discussion**

The instrument was only tested with participants from 2 countries. Consider adding this as another limitation.

Lines 446-448 – Present this earlier in the Discussion, before the final paragraph. The final paragraph should be reserved for a brief overview and/or next steps.
