# Supplementary material for: The development of the ScoPeO-Kids: A new measure of quality of life for children living in contexts of vulnerability
Source: PLOS Glob Public Health. 2026 Sep 9;6(9):e0007247. doi: 10.1371/journal.pgph.0007247 (PMC13557343; doi:10.1371/journal.pgph.0007247)
Supplement: Supplementary file 8 [file pgph.0007247.s008.docx]

## **Overall Assessment**

This is a **strong and relevant manuscript** addressing an important gap in measuring quality of life (QoL) among vulnerable children, particularly in low-resource and humanitarian contexts. The mixed-methods approach and integration of child perspectives are notable strengths. However, the manuscript would benefit from **greater conceptual clarity, methodological transparency, and critical reflection**, especially given its intended contribution to global public health measurement tools.

## **Major Comments**

### 1. **Conceptual Clarity of QoL**

- The manuscript explicitly states that **no a priori definition of QoL was used** (p.6–7), which is methodologically interesting but problematic.
- This weakens conceptual grounding and makes it difficult to assess:
  - Construct validity
  - Theoretical alignment with existing QoL frameworks
- Recommendation:
  - Provide a **working definition of QoL emerging from the study**, even if inductive.
  - Clarify how frameworks (e.g., Maslow, Round et al.) concretely informed item development.

### 2. **Population and “Vulnerability” Framing**

- The definition of “vulnerable children” is broad (poverty, disability, conflict, etc.) (p.6).
- This risks:
  - Overgeneralization
  - Masking heterogeneity across contexts
- Recommendation:
  - Clarify whether the tool is intended to be:
    - Universally applicable across vulnerable groups, or
    - Context-specific
  - Reflect more critically on **intersectionality and structural determinants**.

### 3. **Sampling and Representativeness**

- The study includes:
  - 574 children across Thailand, Bangladesh, Pakistan (p.19)
- However:
  - Sampling is **not clearly representative**
  - Exclusion of children with communication disabilities is acknowledged (p.25), but implications are underdeveloped
- Recommendation:
  - Discuss **selection bias and inclusivity limitations** more critically
  - Clarify recruitment procedures and any power dynamics (e.g., NGO-led recruitment)

### 4. **Use of Translation and Cross-Cultural Validity**

- Heavy reliance on translators during focus groups (p.11–12, p.25)
- Potential risks:
  - Meaning distortion
  - Loss of cultural nuance
- Recommendation:
  - Expand discussion on:
    - Translation validation processes
    - Cultural adaptation strategies
  - Consider referencing cross-cultural validation standards

### 5. **Rasch Analysis Interpretation**

- The Rasch analysis is a strength but needs clearer explanation:
  - 17 items misfit (p.20)
  - All 43 items initially had disordered thresholds (p.21)
- These are significant issues but not critically unpacked.
- Recommendation:
  - Explain:
    - Why misfit occurred
    - Implications for construct validity
  - Provide clearer justification for item removal beyond statistical fit

### 6. **Response Scale Revision**

- Transition from Likert (5-point) to frequency (4-point) scale (p.22–23)
- This is a major conceptual shift but insufficiently justified.
- Recommendation:
  - Clarify:
    - Why frequency is more appropriate for children
    - Implications for comparability with other QoL tools

### 7. **Ethical Considerations**

- Ethics approval and consent are well stated (p.8)
- However:
  - Limited discussion of:
    - Power dynamics with children
    - Safeguarding in vulnerable settings
- Recommendation:
  - Strengthen ethical reflection, especially for:
    - Children in refugee camps
    - Emotional risks during discussions

## **Minor Comments**

### Writing & Clarity

- Some grammatical inconsistencies:
  - e.g., “with led to the deletion…” (Abstract, p.1 & p.5)
- Repetition between abstract and results sections
- Recommendation:
  - Careful proofreading and tightening of language

### Structure

- The Results section could be more concise:
  - Tables are useful but sometimes overly dense (e.g., Table 1, p.15)
- Recommendation:
  - Highlight key findings more sharply

### Data Availability Statement

- Data is not publicly available (p.2)
- This partially conflicts with PLOS open data expectations
- Recommendation:
  - Clarify access process more transparently

## **Contribution to the Field**

**Strengths:**

- Child-centered methodology (focus groups, cognitive debriefing)
- Inclusion of humanitarian settings
- Development of a potentially adaptable QoL tool

**However, to strengthen impact:**

- Position more clearly against **existing QoL tools** (e.g., PedsQL, KIDSCREEN)
- Emphasize **what is truly novel**:
  - Safety domain
  - Contextualized items (e.g., food insecurity, autonomy)

## **Suggested Decision**

**Minor revisions required**

The manuscript has strong potential, but requires:

- Deeper theoretical grounding
- Clearer methodological justification
- Stronger critical and ethical reflection
