# Supplementary material for: The development of the ScoPeO-Kids: A new measure of quality of life for children living in contexts of vulnerability
Source: PLOS Glob Public Health. 2026 Sep 9;6(9):e0007247. doi: 10.1371/journal.pgph.0007247 (PMC13557343; doi:10.1371/journal.pgph.0007247)
Supplement: Supplementary file 9 [file pgph.0007247.s009.docx]

**Peer review comments for PGPH-D-25-02405R2**

1. Consider clarifying how you distinguish QoL from closely related constructs like well-being, mental health, functioning, and protection outcomes. Additionally, if the measure is meant to evaluate “enabling environments,” it would be helpful to explicitly demonstrate the theory of change linking environment to QoL and long-term outcomes.
2. Regarding study population and context specificity, in addition to age ranges, gender, etc., did the study account for unaccompanied minors or the length of displacement? Also, please explain if/how the researcher accounted for heterogeneity across camp and IDP community settings, such as governance structures, humanitarian actors, cultural norms, etc. Is the expectation that a single measure is to be valid across these contexts?
3. Regarding the child-centered methodology, I appreciate the use of participatory child-centered approaches that allow young children to define what QoL means to themselves, rather than imposing adult-derived domains.
4. What adaptations, if any, were made for those with trauma? For children and parents with severe hearing, attention, or communication disabilities? I did see that the latter were excluded from the study. Doesn’t this favor the underrepresentation of this doubly vulnerable category? Wouldn’t the absence of disability-specific QoL domains render them effectively invisible in the QoL measure? These should be clarified in the ethics section.
5. Noting potential ethics concerns that research with displaced children entails, please explain how informed consent/assent was obtained in contexts where guardianship was unclear? In the interviews and focus group discussions, what safeguards were in place to prevent distress or re-traumatization? What referral pathways existed if distress or protection concerns emerged during data collection? These should be clarified in the ethics section as well.
6. Similarly, please share considerations, if any, for risk of harm through measurement. Could labeling children with “low QoL” have unintended consequences (e.g., stigma, exclusion, misallocation of aid)? How might one mitigate the risk of this tool being misused by agencies or donors?
7. Considering the challenges mentioned in translation during interviews, were local idioms of distress and well-being incorporated? How did the authors ensure the measure was not embedding culturally biased assumptions about what constitutes a “good life”?
8. I am unclear about how QoL scores will be interpreted. Are there cutoffs, or is it a continuous index? What decisions should (and should not) be made based on these scores?
9. QoL in refugee settings is highly volatile; for example, a food distribution delay, security incident, or relocation can shift responses overnight. Are the authors treating QoL as a stable construct when it may be situational and time-sensitive? What is the recall period, and is it appropriate for such unstable environments? Have authors considered ecological momentary approaches or repeated short-interval measures?
10. Additionally, children in prolonged displacement may recalibrate what they consider “normal” or “good.” How do the authors address response shift, where children report acceptable QoL despite objectively poor conditions? Are evaluators at risk of underestimating deprivation because children have adapted expectations?
11. In camps and vulnerable settings, children are often aware that adults with clipboards are linked (directly or indirectly) to aid. What steps were taken to reduce courtesy bias or “gratitude bias”, and to minimize perceived authority/ power dynamics or social desirability? Is there evidence that children felt safe giving negative responses?
12. How were discrepancies in QoL measure via proxies handled analytically and conceptually? What happens when a parent/guardian reports high QoL but the child reports distress (or vice versa)?
13. Considering the reliance of QoL tools on subjective perception, how do the authors avoid conflating resilience with acceptable living standards, or in scenarios where refugees are confined to a camp?
14. How does the QoL measure align or intersect with established protection frameworks used by actors like UNICEF or the Interagency Standing Committee? Are critical protection risks (violence, exploitation, early marriage, child labor) treated as QoL domains, covariates, or excluded entirely?
15. Who owns the data collected from vulnerable children? Will results be shared back with communities and participants in an accessible way? How would QoL scores be linked to actual decision-making? What are the potential implications of high vs. low scores on, say, programmatic input, funding cycles?
16. Line 279 -285: Unclear about how “themes being chosen and grouped and converted to questionnaire.” It would be helpful to outline/ illustrate how these were arrived at and demonstrate with an example how this was achieved.
17. Lines 306 – 327 seem a bit obscure and hard to understand for someone unfamiliar with the Rasch model and how these analyses were arrived at. Consider rewriting in plain(er) language.
18. Beyond stratification and “control for” gender and age, are there gender-specific constructs (e.g., mobility restrictions, menstrual hygiene, safety concerns) embedded in the measure?
19. Regarding feasibility in humanitarian settings, can this QoL tool actually be used in camps? How long does it take to administer? Does it require trained enumerators or specialists? Consider realistic implementation burden on frontline staff, required training, in high-turnover humanitarian settings. Can it be integrated into existing monitoring systems used by organizations like UNICEF or UNHCR?
20. Since the stated research goal is to “evaluate interventions,” is there evidence that the measure is responsive to change over time? Can it detect meaningful differences attributable to interventions, rather than just background variability?
21. Have the authors considered how the measure might distort priorities once tied to funding or evaluation? Could organizations “game” the metric to show improvement without meaningful change?
22. Regarding enabling environments, does the tool differentiate between home/shelter, school, community spaces, and informal environments? Are safety and freedom of movement measured in context-specific ways (e.g., within vs. outside the camp)?
23. What are the limits of generalization beyond the specific camps, communities, or regions studied?
