# Supplementary material for: The development of the ScoPeO-Kids: A new measure of quality of life for children living in contexts of vulnerability
Source: PLOS Glob Public Health. 2026 Sep 9;6(9):e0007247. doi: 10.1371/journal.pgph.0007247 (PMC13557343; doi:10.1371/journal.pgph.0007247)
Supplement: Supplementary file 10 [file pgph.0007247.s010.docx]

Montreal, June 15^th^, 2026

Dear editors and reviewers,

We thank you again for your time and care in reviewing our manuscript. We are very grateful to the reviewers for their detailed and constructive feedback during the revision process, which has significantly improved the article.

We note, however, that several comments raised in earlier rounds appear to have been satisfactorily addressed, as acknowledged by Reviewer #5 and that several comments raised in this round revisit points that had already been addressed in earlier revisions (models, Rasch, etc.). At this stage and considering the numerous modifications already made in response to the reviewers’ suggestions, we feel that further changes may risk moving the manuscript away from its original objectives and central contribution.

We therefore respectfully submit this version of the manuscript as final, and hope that it will be suitable for publication. We would, of course, be glad to make any minor adjustments that remain.

Thank you once again for taking it into consideration.

Best regards,

Johanne Higgins on behalf of co-authors

Full professor

École de réadaptation

Faculty of Medicine

Montréal QC, Canada, H3C 3J7

Researcher, Centre de recherche interdisciplinaire en réadaptation du Montréal métropolitain (CRIR)

Site de l’Institut universitaire sur la réadaptation en déficience physique du CIUSSS Centre-Sud-de-l'Île-de-Montréal.
