# Supplementary material for: The development of the ScoPeO-Kids: A new measure of quality of life for children living in contexts of vulnerability
Source: PLOS Glob Public Health. 2026 Sep 9;6(9):e0007247. doi: 10.1371/journal.pgph.0007247 (PMC13557343; doi:10.1371/journal.pgph.0007247)
Supplement: Peer review history

# Peer review: Decision Letter 0

**DOI:** 10.1371/journal.pgph.0007247.r001

**Authors:** Steve Zimmerman

17 Nov 2025

PGPH-D-25-02405

The development of the ScoPeO-Kids: a new measure of quality of life for children living in contexts of vulnerability.

PLOS Global Public Health

Dear Dr. Higgins,

Thank you for submitting your manuscript to PLOS Global Public Health. After careful consideration, we feel that it has merit but does not fully meet PLOS Global Public Health’s publication criteria as it currently stands. Therefore, we invite you to submit a revised version of the manuscript that addresses the points raised during the review process.

The manuscript has been evaluated by two reviewers, and their comments are available below.

Could you please carefully revise the manuscript to address all comments raised?

Please submit your revised manuscript by Dec 29 2025 11:59PM. If you will need more time than this to complete your revisions, please reply to this message or contact the journal office at globalpubhealth@plos.org. When you're ready to submit your revision, log on to https://www.editorialmanager.com/pgph/ and select the 'Submissions Needing Revision' folder to locate your manuscript file.

Please include the following items when submitting your revised manuscript:

- A rebuttal letter that responds to each point raised by the editor and reviewer(s). You should upload this letter as a separate file labeled 'Response to Reviewers'.
- A marked-up copy of your manuscript that highlights changes made to the original version. You should upload this as a separate file labeled 'Revised Manuscript with Track Changes'.
- An unmarked version of your revised paper without tracked changes. You should upload this as a separate file labeled 'Manuscript'.

Guidelines for resubmitting your figure files are available below the reviewer comments at the end of this letter.

We look forward to receiving your revised manuscript.

Kind regards,

Steve Zimmerman, PhD

PLOS Staff Editor

Journal Requirements:

1. Please include a complete copy of PLOS’ questionnaire on inclusivity in global research in your revised manuscript. Our policy for research in this area aims to improve transparency in the reporting of research performed outside of researchers’ own country or community. The policy applies to researchers who have travelled to a different country to conduct research, research with Indigenous populations or their lands, and research on cultural artefacts. The questionnaire can also be requested at the journal’s discretion for any other submissions, even if these conditions are not met.  Please find more information on the policy and a link to download a blank copy of the questionnaire here: https://journals.plos.org/globalpublichealth/s/best-practices-in-research-reporting. Please upload a completed version of your questionnaire as Supporting Information when you resubmit your manuscript.

If the reviewer comments include a recommendation to cite specific previously published works, please review and evaluate these publications to determine whether they are relevant and should be cited. There is no requirement to cite these works unless the editor has indicated otherwise.

Additional Editor Comments (if provided):

[Note: HTML markup is below. Please do not edit.]

Reviewers' comments:

Reviewer's Responses to Questions

**Comments to the Author**

1. Does this manuscript meet PLOS Global Public Health’s publication criteria?

Reviewer #1: Yes

Reviewer #2: Partly

**********

2. Has the statistical analysis been performed appropriately and rigorously?

Reviewer #1: Yes

Reviewer #2: No

**********

3. Have the authors made all data underlying the findings in their manuscript fully available (please refer to the Data Availability Statement at the start of the manuscript PDF file)?

The PLOS Data policy

Reviewer #1: Yes

Reviewer #2: No

**********

4. Is the manuscript presented in an intelligible fashion and written in standard English?

Reviewer #1: Yes

Reviewer #2: Yes

**********

Reviewer #1: Thank you for the opportunity to review the manuscript, “The development of the ScoPeO-Kids: A new measure of quality of life for children living in contexts of vulnerability.” The purpose of this project was to develop an instrument to measure quality of life for children living in situations of vulnerability. This was an interesting paper, but revisions must be made before the paper is suitable for publication. Most importantly, more details are needed throughout the Method and Results sections. Please see recommendations below:

Abstract

Reword the sentence on lines 34-35 beginning with “The pool of items…” to read “The instrument was administered to….”

Consider removing lines 37-41.

Introduction

Slow down a bit. The first paragraph jumps right into predictors of quality of life. Take some time to set this up for the reader.

Line 94 – change “to the focus groups” to “of the focus groups…”

Line 97 – data “were”

Lines 119-122 – take some time to briefly provide an overview of the frameworks and why they were particularly relevant to your project.

Method

Line 140 – provide a definition of slums and communities with a high prevalence of poverty

Provide details of participants in the Children Focus Groups and Parents Focus Groups sections (p. 8-9) rather than in the Results.

Children Focus Groups section

• Line 154 – provide a rationale for the use of these age groups

• Describe how the protocols were differentiated based on age. Provide protocols as a table or supplemental material.

Begin a new paragraph, starting with “All focus group discussions were audio-recorded….” (line 184).

Provide additional details regarding thematic analysis (lines 186-188). Example, how many coders analyzed data? What did the process look like.

Lines 197-201 – How was demographic info provided (pencil and paper or on a tablet or computer?). Describe any help provided to younger participants.

Lines 222-223 – Provide more information of the 25 children who participated in the cognitive debriefing exercise (e.g., how many from each country, ages, genders)

Results

Consider providing a table outlining key characteristics of QoL measures identified in the literature review.

Workshop with HI should be moved to the Method section. Further details should be provided regarding workshop activities.

Line 247 – different word for “reuniting.”

Consider defining abbreviations in Table 2.

Line 270-271 – revise to read “Finally, parents associated the safety of the….”

Table 3 – Additional details are needed. Provide some context for participants who said each of the quotes.

Line 280 – Who compared findings from the workshop? How?

Lines 291- 293 – Define “younger age” and “low reading skills.” How were reading skills determined?

Table 5 – Label the right column “n (%)” and remove “no. (%)” from each row.

Line 307 – More information is needed on the first factor (eigenvalue 9.6). Which items loaded on this factor?

Clarification is needed for lines 323 – 326. This is confusing as written.

Figure 1 seems to be missing.

Discussion

The instrument was only tested with participants from 2 countries. Consider adding this as another limitation.

Lines 446-448 – Present this earlier in the Discussion, before the final paragraph. The final paragraph should be reserved for a brief overview and/or next steps.

Reviewer #2: Thank you for the opportunity to read and review this interesting and well written article. The instrument looks also very good and is precious, as good scales to assess the lived experience of children are difficult to find. However, I am sorry, the method section needs to be more structured, the result section too. Some of the requirements of the Rasch model were not tested (i.e. local item dependencies). I would suggest to maybe search for a few Rasch analyses to see how to describe the methods and discuss results – check maybe also Tennant and Conaghan 2007.

Abstract:

Line 40 – correct the grammar of the sentence "This analysis combined with cognitive debriefing sessions with led to the deletion of 23 items. "

Line 48: The impacts – without s.

Line 100 - participants were recruited in 2017, like 8 years ago. What is the timing of this study, data collection etc. Does the setting of 2017 still apply today? A lot has changed with the new technologies and maybe also as consequence to the covid pandemia (I am thinking of life in refugee camps).

Lines 119-122 – Please rephrase this sentence: The models proposed by Round and colleagues (13) and Fagerlind schematisation, completed by Maslow’s goals or needs perspective (14), were used as frameworks to support the reflexion behind the development of the ScoPeO-kids. Ex. – Several frameworks supported the reflexion …

Line 149: pooled by domains?

Line 200 : Bangladesh

Line 207: I am unsure if the PCA was done on the data or on the residuals of the Rasch analysis. It reads like the PCA was done before the Rasch analysis in SAS. I don't think that that PCA is valid approach on the raw, ordered data, or maybe with polychoric correlations or strategy to have interval scaled data.

In RUMM2030, PCA of residuals can be tested to decide if a set of items is unidimensional. I would rather suggest making the test of dimensionality, and also the selection of items in RUMM2030 - and check the eigenvalues and component loadings there instead of a separate analysis.

Line 209: Fit was assessed to verify fit… Please rephrase following sentence "Overall fit and individual item fit were assessed to verify adequate fit of the items and their response options to the model." The item fit does not say anything about the threshold ordering.

Line 212 – "Rasch analyses were used to detect biases "– suggestion to rather write for example "An analysis of differential item functioning allowed to investigate lack of invariance within items for groups of age and country of origin.

Line 214: "Monotonocity was also verified through fit statistics" – Monotonicity is not verified with the fit statistics. You could find disordered thresholds in items with good fit as well as, reversely, misfitting items with ordered thresholds. Monotonicity is investigated by means of the estimates of the response difficulty thresholds and their ordering.

Line 215 – here (and in this entire section), the information could be more specific. Reliability is assessed with the person separation index, where values above 0.85 are considered good fit and above 0.7 as sufficient… or is interpreted similarly to Cronbach alpha… Later the authors write about person separation, so it would make sense to mention it in the method section already.

Line 215 could the authors say more about the response options for the selected questions already here?

Line 306 – it is quite uncommon to use the Kaiser criterium (1st eigen <1) to decide on the dimensionality of a scale - when performin PCA on residuals... Based on Linacre 1st eigenvalues > 2 are strong indication for multidimensionality or Smith and Miao suggest a second eigenvalue > 1.4.

Line 225 – were the children interviewed? It surprises me that children of age 5 are already completing a questionnaire. How were the interviews done for the children that could not read?

Line 288 – the information about the response options could be provided earlier.

Line 304 – what does IDP stand for? (make sure that all acronyms can be understood)

Line 310 – With a a PCA, you would have component loadings (and not factors) and eigenvalues. You would have as many components as items in the scale. You would first observe which items load positively or negatively on the first component. You could also plot the values of the first against the second component and see which items cluster etc…

Line 313 . "Analysis of the 43 items indicated that not all items met the requirements of the Rasch model". This section is confusing as it first mentions the items (that do not meet requirements of the Rasch model). Than, it is about the model fit. Also requirements of the Rasch model are not only about the item fit – threshold ordering, local independence and absence of DIF are also important requirements.

Line 317 – please round it e.g. p-value < 0.001.

Line 319 – the PSI is very likely to be inflated - because of remaining dependencies and multidimensionality.

Line 323 - Please explain what it means to have a big score versus a small score on the scale.

Line 349 – I am glad to hear that you could reorder the thresholds just through collapsing options 1 and 2.

Line 364 – A response scale that mixes perspectives can be confusing and may be a limitation. Your response scale suggests as option not often/a little and often/ a lot. (I am not sure about the phrasing of the items but - "things" can happen often but with little effect also and reversely can happen rarely but with big effect. In that sense, a unidimensional response mode would be less ambiguous (intensity even better than frequency). For example items on conflicts at home - if seldom but when, than very violent - what response is it not often or a lot?.

In this section, I miss an analysis of local item dependencies (LID). It would be interesting to know how the multidimensionality and LID are connected, if the selection of items show groups of items. Absence of local item dependencies would provide unbiased estimate of reliability.

The discussion is interesting, but many paragraphs are too closely describing results that could have been or were mentioned previously. In that sense the discussion section needs some work in deciding what is a discussion point or a result. Also this discussion could benefit of more information regarding the implication of this study, the advantage of this tool versus existing tools.

Line 446 – Regarding the conclusion – the authors mention that the ScoPeO-kids tool is needed because

two domains are specific to children in slums or low socio-economic communities (safety and autonomy/self-realization). How are these items specific to this population? Are they not important for all children? Do other tools omit to assess these aspects and for this reason the ScoPeO-kid is a plus?

**********

what does this mean?). If published, this will include your full peer review and any attached files.

**Do you want your identity to be public for this peer review?**  If you choose “no”, your identity will remain anonymous but your review may still be made public.

For information about this choice, including consent withdrawal, please see our Privacy Policy

Reviewer #1: No

Reviewer #2: No

**********

[NOTE: If reviewer comments were submitted as an attachment file, they will be attached to this email and accessible via the submission site. Please log into your account, locate the manuscript record, and check for the action link "View Attachments". If this link does not appear, there are no attachment files.]

Attachment Submitted filename: QoL_PLOS Review_Nov_2025.docx

# Author comment: Author response to Decision Letter 1

**DOI:** 10.1371/journal.pgph.0007247.r002

19 Feb 2026

Attachment Submitted filename: Response to Reviewers.pdf

# Peer review: Decision Letter 1

**DOI:** 10.1371/journal.pgph.0007247.r003

**Authors:** Md. Alamgir Hossain

8 Mar 2026

PGPH-D-25-02405R1

The development of the ScoPeO-Kids: a new measure of quality of life for children living in contexts of vulnerability.

PLOS Global Public Health

Dear Dr. Johanne Higgins,

Thank you for submitting your manuscript to PLOS Global Public Health. After careful consideration, we feel that it has merit but does not fully meet PLOS Global Public Health’s publication criteria as it currently stands. Therefore, we invite you to submit a revised version of the manuscript that addresses the points raised during the review process.

Please submit your revised manuscript by 25th March, 2026. If you will need more time than this to complete your revisions, please reply to this message or contact the journal office at globalpubhealth@plos.org. When you're ready to submit your revision, log on to https://www.editorialmanager.com/pgph/ and select the 'Submissions Needing Revision' folder to locate your manuscript file.

Please include the following items when submitting your revised manuscript:

- A letter that responds to each point raised by the editor and reviewer(s). You should upload this letter as a separate file labeled 'Response to Reviewers'.
- A marked-up copy of your manuscript that highlights changes made to the original version. You should upload this as a separate file labeled 'Revised Manuscript with Track Changes'.
- An unmarked version of your revised paper without tracked changes. You should upload this as a separate file labeled 'Manuscript'.

Guidelines for resubmitting your figure files are available below the reviewer comments at the end of this letter.

We look forward to receiving your revised manuscript.

Kind regards,

Md. Alamgir Hossain, M.S.S., M.P.H.

Academic Editor

PLOS Global Public Health

Journal Requirements:

If the reviewer comments include a recommendation to cite specific previously published works, please review and evaluate these publications to determine whether they are relevant and should be cited. There is no requirement to cite these works unless the editor has indicated otherwise.

Please review your reference list to ensure that it is complete and correct. If you have cited papers that have been retracted, please include the rationale for doing so in the manuscript text, or remove these references and replace them with relevant current references. Any changes to the reference list should be mentioned in the rebuttal letter that accompanies your revised manuscript. If you need to cite a retracted article, indicate the article’s retracted status in the References list and also include a citation and full reference for the retraction notice.

Additional Editor Comments (if provided):

[Note: HTML markup is below. Please do not edit.]

Reviewers' comments:

Reviewer's Responses to Questions

**Comments to the Author**

Reviewer #3: (No Response)

Reviewer #4: (No Response)

**********

publication criteria?

Reviewer #3: Yes

Reviewer #4: Partly

**********

3. Has the statistical analysis been performed appropriately and rigorously?

Reviewer #3: Yes

Reviewer #4: I don't know

**********

4. Have the authors made all data underlying the findings in their manuscript fully available (please refer to the Data Availability Statement at the start of the manuscript PDF file)?

The PLOS Data policy

Reviewer #3: Yes

Reviewer #4: No

**********

5. Is the manuscript presented in an intelligible fashion and written in standard English?

Reviewer #3: Yes

Reviewer #4: Yes

**********

Reviewer #3: Although the introduction provides a strong rationale for the study, it could better articulate the theoretical frameworks that inspired the study. Frameworks such as Maslow’s hierarchy are mentioned, but their specific application to the development of this measure is not clear. How did Maslow’s theory of needs help shape the selection of domains such as safety or social well-being? A clearer link of theory to the development of the items would strengthen the conceptual foundations.

The rationale for excluding specific items (e.g., “balance between play/leisure and work”) and the decision-making process for prioritizing items are not fully explained in the introduction. This raises questions about the subjective nature of item selection. Therefore, explain how the input from different stakeholders (e.g., parents, children, professionals) was weighed and balanced.

While a Rasch analysis is performed to confirm the psychometric properties of the instrument, the results section lacks a comprehensive explanation of the statistical method. For example, the explanation of “item-attribute interaction” and “fit residuals” is too technical for readers who may not be familiar with Rasch analysis.

In the methodology section, provide more information about the inclusion/exclusion criteria for participants, particularly with regard to children with disabilities. Clarify whether specific steps were taken to ensure adequate representation of the diversity within this group (e.g., different levels of disability severity).

I would like to kindly suggest citing the following articles, which I believe could provide additional context and reinforce certain points within your manuscript:

Article 1: Kazemi Karyani A, Rezaei S, Karami Matin B, Amini S. Poor health-related quality of life in Iran: decomposition analysis of affecting factors. International Journal of Human Rights in Healthcare. 2019 Feb 21;12(1):28-37.

Comment: "The quality of life (QoL) for individuals in vulnerable populations can be significantly impacted by factors such as poverty, disability, and environmental conditions, making it essential to consider these contextual elements when designing and evaluating QoL measures."

Placement in Manuscript: This comment can be inserted after the sentence ending with "need for a tool capable of measuring the outcomes of such interventions on the QoL of children regardless of their gender, age or disability status."

Article 2: Nazari J, Jafari K, Chegini M, Maleki A, MirShafiei P, Alimohammadi A, Kazemzadeh Y, Mikaeliyan R, Amini S. Physical and mental growth and development in children with congenital hypothyroidism: a case–control study. Orphanet journal of rare diseases. 2021 Sep 23;16(1):393.

Comment: "Children’s physical and mental growth, as well as their emotional well-being, are deeply interconnected and influenced by a range of social, environmental, and psychological factors that should be assessed holistically in any quality of life measure."

Placement in Manuscript: This comment can be inserted after the sentence ending with "understanding the factors that influence the QoL in vulnerable children is of utmost importance to deliver evidence-based interventions."

Reviewer #4: This manuscript addresses an important gap in the measurement of child well-being in vulnerable contexts and presents a promising new instrument. With greater clarity regarding the conceptual framework, methodological procedures, and cross-cultural validation, the manuscript has the potential to make a valuable contribution to global public health research and practice.

This study potentially fills a gap in how we measure global public health, especially since many existing QoL tools were developed in high-income countries and may not accurately reflect the experiences of children facing poverty, displacement, disability, or humanitarian crises. Developing context-specific tools to measure child well-being in vulnerable populations is critical in global health. The manuscript is both timely and meaningful, showcasing several valuable strengths. The multi-stage instrument development process, including the inclusion of both children and parents helps ensure that lived experiences are reflected well in the instruments. The use of Rasch modelling demonstrates attention to measurement validity. Lastly, collaboration with NGO has enhanced the operational relevance of the work. To strengthen it further, some parts related to the conceptual framework, methodological details, and interpretation could be clarified and made more scientifically sound.

1. Conceptual Framework

The manuscript references several conceptual perspectives on quality of life, yet it is not entirely clear how these frameworks informed the development of the final domains. More explicit explanation of the relationship between the initial conceptual framework and the final five-domain structure would strengthen the interpretability as well as coherence.

2. Scope

How the authors defined ‘vulnerability’ should be made clearer. A clearer definition of the target population would help readers understand the instrument's contexts of applicability.

3. Sampling and Recruitment

More detail regarding participant recruitment would improve transparency, e.g. how children and caregivers were identified and recruited through partner organisations, and whether there might have been selection bias.

4. Psychometric Reporting

It would also be helpful to include key item statistics (e.g., item difficulty and fit indices) in a supplementary table. Additionally, the implications of the observed ceiling effect should be discussed in greater detail, notably regarding the instrument’s sensitivity to detect change in intervention evaluations.

5. Cross-Cultural Validity and Translation

The instrument was administered across several countries and languages, yet the author provides limited detail on the translation and cultural adaptation procedures, which could be important for increasing confidence in the instrument's applicability.

Lastly, the discussion could also further explain how the instrument should be used operationally, including how scores should be interpreted in program evaluation contexts.

**********

what does this mean?). If published, this will include your full peer review and any attached files.

**Do you want your identity to be public for this peer review?**  If you choose “no”, your identity will remain anonymous but your review may still be made public.

For information about this choice, including consent withdrawal, please see our Privacy Policy

Reviewer #3: No

Reviewer #4: No

**********

[NOTE: If reviewer comments were submitted as an attachment file, they will be attached to this email and accessible via the submission site. Please log into your account, locate the manuscript record, and check for the action link "View Attachments". If this link does not appear, there are no attachment files.]

# Author comment: Author response to Decision Letter 2

**DOI:** 10.1371/journal.pgph.0007247.r004

21 Apr 2026

Attachment Submitted filename: Response to reviewers 2.pdf

# Peer review: Decision Letter 2

**DOI:** 10.1371/journal.pgph.0007247.r005

**Authors:** Md. Alamgir Hossain

8 May 2026

PGPH-D-25-02405R2

The development of the ScoPeO-Kids: a new measure of quality of life for children living in contexts of vulnerability.

PLOS Global Public Health

Dear Dr. Higgins,

Thank you for submitting your manuscript to PLOS Global Public Health. After careful consideration, we feel that it has merit but does not fully meet PLOS Global Public Health’s publication criteria as it currently stands. Therefore, we invite you to submit a revised version of the manuscript that addresses the points raised during the review process.

Please submit your revised manuscript by 15/06/2026. If you will need more time than this to complete your revisions, please reply to this message or contact the journal office at globalpubhealth@plos.org. When you're ready to submit your revision, log on to https://www.editorialmanager.com/pgph/ and select the 'Submissions Needing Revision' folder to locate your manuscript file.

Please include the following items when submitting your revised manuscript:

- A letter that responds to each point raised by the editor and reviewer(s). You should upload this letter as a separate file labeled 'Response to Reviewers'.
- A marked-up copy of your manuscript that highlights changes made to the original version. You should upload this as a separate file labeled 'Revised Manuscript with Track Changes'.
- An unmarked version of your revised paper without tracked changes. You should upload this as a separate file labeled 'Manuscript'.

Guidelines for resubmitting your figure files are available below the reviewer comments at the end of this letter.

As the corresponding author, your ORCID iD is verified in the submission system and will appear in the published article. PLOS supports the use of ORCID, and we encourage all coauthors to register for an ORCID iD and use it as well. Please encourage your coauthors to verify their ORCID iD within the submission system before final acceptance, as unverified ORCID iDs will not appear in the published article. *Only*  the individual author can complete the verification step; PLOS staff *cannot*  verify ORCID iDs on behalf of authors.

We look forward to receiving your revised manuscript.

Kind regards,

Md. Alamgir Hossain, M.S.S., M.P.H.

Academic Editor

PLOS Global Public Health

Journal Requirements:

If the reviewer comments include a recommendation to cite specific previously published works, please review and evaluate these publications to determine whether they are relevant and should be cited. There is no requirement to cite these works unless the editor has indicated otherwise.

Please review your reference list to ensure that it is complete and correct. If you have cited papers that have been retracted, please include the rationale for doing so in the manuscript text, or remove these references and replace them with relevant current references. Any changes to the reference list should be mentioned in the rebuttal letter that accompanies your revised manuscript. If you need to cite a retracted article, indicate the article’s retracted status in the References list and also include a citation and full reference for the retraction notice.

Additional Editor Comments (if provided):

[Note: HTML markup is below. Please do not edit.]

Reviewers' comments:

Reviewer's Responses to Questions

**Comments to the Author**

Reviewer #5: All comments have been addressed

Reviewer #6: (No Response)

**********

publication criteria?

Reviewer #5: Yes

Reviewer #6: Partly

**********

3. Has the statistical analysis been performed appropriately and rigorously?

Reviewer #5: N/A

Reviewer #6: I don't know

**********

4. Have the authors made all data underlying the findings in their manuscript fully available (please refer to the Data Availability Statement at the start of the manuscript PDF file)?

The PLOS Data policy

Reviewer #5: Yes

Reviewer #6: No

**********

5. Is the manuscript presented in an intelligible fashion and written in standard English?

Reviewer #5: Yes

Reviewer #6: Yes

**********

Reviewer #5: Good Study

Reviewer #6: (No Response)

**********

what does this mean?). If published, this will include your full peer review and any attached files.

**Do you want your identity to be public for this peer review?**  If you choose “no”, your identity will remain anonymous but your review may still be made public.

For information about this choice, including consent withdrawal, please see our Privacy Policy

Reviewer #5: **Yes:**  Tadiwanashe Burukai-Matutu | Creative Feminist Consulting

Reviewer #6: **Yes:**  Dr. Floramae Esapebong-Ray

**********

[NOTE: If reviewer comments were submitted as an attachment file, they will be attached to this email and accessible via the submission site. Please log into your account, locate the manuscript record, and check for the action link "View Attachments". If this link does not appear, there are no attachment files.]

Attachment Submitted filename: Minor Revisions.docx

Attachment Submitted filename: Peer review comments for PGPH-D-25-02405R2.docx

# Author comment: Author response to Decision Letter 3

**DOI:** 10.1371/journal.pgph.0007247.r006

15 Jun 2026

Attachment Submitted filename: Response to reviewers 3.docx

# Peer review: Decision Letter 3

**DOI:** 10.1371/journal.pgph.0007247.r007

**Authors:** Md. Alamgir Hossain

16 Aug 2026

The development of the ScoPeO-Kids: a new measure of quality of life for children living in contexts of vulnerability.

PGPH-D-25-02405R3

Dear Dr. Johanne Higgins,

We are pleased to inform you that your manuscript 'The development of the ScoPeO-Kids: a new measure of quality of life for children living in contexts of vulnerability.' has been provisionally accepted for publication in PLOS Global Public Health.

Before your manuscript can be formally accepted you will need to complete some formatting changes, which you will receive in a follow up email. A member of our team will be in touch with a set of requests.

Please note that your manuscript will not be scheduled for publication until you have made the required changes, so a swift response is appreciated.

IMPORTANT: The editorial review process is now complete. PLOS will only permit corrections to spelling, formatting or significant scientific errors from this point onwards. Requests for major changes, or any which affect the scientific understanding of your work, will cause delays to the publication date of your manuscript.

If your institution or institutions have a press office, please notify them about your upcoming paper to help maximize its impact. If they'll be preparing press materials, please inform our press team as soon as possible -- no later than 48 hours after receiving the formal acceptance. Your manuscript will remain under strict press embargo until 2 pm Eastern Time on the date of publication. For more information, please contact globalpubhealth@plos.org.

Thank you again for supporting Open Access publishing; we are looking forward to publishing your work in PLOS Global Public Health.

Best regards,

Md. Alamgir Hossain, M.S.S., M.P.H.

Academic Editor

PLOS Global Public Health

***********************************************************

Reviewer Comments (if any, and for reference):

Reviewer's Responses to Questions

**Comments to the Author**

Reviewer #7: All comments have been addressed

Reviewer #8: (No Response)

**********

publication criteria?

Reviewer #7: Yes

Reviewer #8: Partly

**********

3. Has the statistical analysis been performed appropriately and rigorously?

Reviewer #7: Yes

Reviewer #8: No

**********

4. Have the authors made all data underlying the findings in their manuscript fully available (please refer to the Data Availability Statement at the start of the manuscript PDF file)?

The PLOS Data policy

Reviewer #7: Yes

Reviewer #8: Yes

**********

5. Is the manuscript presented in an intelligible fashion and written in standard English?

Reviewer #7: Yes

Reviewer #8: Yes

**********

Reviewer #7: Recommendation: Minor revision.

1. The conceptualisation of Quality of Life requires more robust justification.

Although several theoretical frameworks are introduced, the manuscript does not clearly articulate the process by which these frameworks were integrated into a unified conceptual model. As a result, the conceptual pathway linking these theories to the development of the final five-domain instrument remains unclear.

2. Greater transparency is required in the description of the qualitative analysis. Several important details are missing, including:

• whether data saturation was achieved;

• how themes were refined into questionnaire domains.

3. Abstract

The abstract should include key psychometric findings. This would provide more value than simply stating that Rasch analyses were conducted.

4. Introduction

The literature review is comprehensive. However, it could more clearly distinguish between:

• health-related quality of life and

• broader quality of life.

These concepts are occasionally used interchangeably.

5. Several minor grammatical inconsistencies remain. Examples include:

• inconsistent capitalisation of Quality of Life

• occasional double hyphenation and repeated wording

Reviewer #8: Although this manuscript has clearly benefited from previous rounds of review, some final concerns remain that should be resolved before it can be approved:

1. Unidimensionality of the final 20-item version is asserted, not demonstrated: For the 43-item pool, the authors properly report both PCA-of-residuals eigenvalues and the more rigorous paired t-test criterion — and by their own account, the t-test criterion failed badly (only 17.84% of item-set comparisons met the ≤5% threshold, "suggesting multidimensionality," lines 456–459). For the final 20-item version, they report only that "the first component explained less than 10% of the variance" (lines 521–524) as evidence of unidimensionality and the t-test check is not repeated. Given that the more sensitive test was the one that flagged the problem in the 43-item version, its absence here is a gap, not a formality. I'd ask the authors to either re-run the paired t-test on the reduced 20-item set or explicitly justify why PCA-of-residuals alone is sufficient at this stage (with the caveat that full validation is pending anyway).

2. Local item dependence is reported for the 43-item pool but not re-checked after item removal. Four item pairs exceeded r=0.30 in the original set (lines 488–490). Since dependence can persist or even worsen after item deletion changes the local structure, this should be re-examined for the final 20 items, or explicitly flagged as deferred to the planned validation study.

3. There is a mismatch between what was psychometrically tested and what is being published as the final instrument. The Rasch analysis (fit, targeting, PSI, DIF) was run on the original 43 items using a 5-point agreement scale, later collapsed to 4 categories. Item wording was then substantially revised (13 of 20 final items rewritten) and the response format was changed altogether (from an agreement scale to a 4-point frequency scale (never / not often / often / always) with a new visual (circles of varying size)) based on cognitive debriefing with only 25 children. None of the reported Rasch statistics (fit residuals, PSI=0.92, targeting mean of 0.95 logits) were re-estimated on this final wording/response format. This is arguably the most important limitation of the paper: the psychometric properties currently reported describe an instrument that isn't quite the one being handed to end users. The authors do note that a follow-up validation study is planned, which is reassuring, but I'd ask that the Discussion state explicitly (not just imply) that the reported Rasch indices apply to the pre-cognitive-debriefing version and that the final 20-item/4-point-frequency instrument's fit has not yet been independently verified.

4. There is an inconsistency in domain count and item allocation: In Line ~536-538 authors wrote: "The final 20-item questionnaire covers 5 domains; physical well-being (3 items), safety (2 items), autonomy and self-realization (5 items) and social well-being (5 items)." That's four domains listed, summing to 15 items, not 20. Elsewhere (Discussion, line ~617–618) the five domains are correctly named as physical well-being, emotional well-being, safety, autonomy/self-realization, and social. This needs to be corrected. Presumably "emotional well-being (5 items)" was dropped from the Results sentence.

5. PSI = 0.92 is very likely inflated, and the paper's own discussion says so, but the reader only gets this caveat once, briefly (line ~605–608). Given that residual multidimensionality and LID were both flagged, and both artificially inflate PSI, this deserves a slightly stronger statement in the Discussion (or Abstract/Conclusion) rather than a single subordinate clause, so downstream users of the tool aren't left thinking 0.92 is a clean estimate of “excellent” reliability, as it is mentioned

6. Number of extreme persons excluded: the text says one extreme-score person was excluded leaving N=573 (Lines ~444–448), but the t-test analysis reports N=566 (extreme persons excluded- Lines 454-456). The discrepancy (573 vs. 566) isn't explained — please clarify how many additional cases were dropped and why.

7. A note is needed explaining why Pakistan wasn't included in the qualitative phase

**********

what does this mean?). If published, this will include your full peer review and any attached files.

**Do you want your identity to be public for this peer review?**  If you choose “no”, your identity will remain anonymous but your review may still be made public.

For information about this choice, including consent withdrawal, please see our Privacy Policy

Reviewer #7: No

Reviewer #8: No

**********
